# IPIP27 Coordinates PtdIns(4,5)P_2_ Homeostasis for Successful Cytokinesis

**DOI:** 10.1016/j.cub.2019.01.043

**Published:** 2019-03-04

**Authors:** Sabrya C. Carim, Khaled Ben El Kadhi, Guanhua Yan, Sean T. Sweeney, Gilles R. Hickson, Sébastien Carréno, Martin Lowe

**Affiliations:** 1 School of Biology, Faculty of Biology, Medicine and Health, University of Manchester, The Michael Smith Building, Oxford Road, Manchester M13 9PT, UK; 2 Cellular Mechanisms of Morphogenesis during Mitosis and Cell Motility Division, Institute for Research in Immunology and Cancer, Université de Montréal, Montréal, QC H3C 3J7, Canada; 3 Department of Biology, University of York, York YO10 5DD, UK; 4 Department of Biology, New York University, New York, NY 10003, USA; 5 Center for Genomics and Systems Biology, New York University Abu Dhabi, Saadiyat Island, Abu Dhabi, United Arab Emirates; 6 Department of Pathology and Cell Biology, Université de Montréal, and Sainte-Justine Hospital Research Center, Montréal, QC H3T 1C5, Canada

**Keywords:** phosphoinositide, PtdIns(4,5)P_2_, OCRL, IPIP27, SH3PX1, BAR domain protein, cytokinesis, actin, endosome

## Abstract

During cytokinesis, an actomyosin contractile ring drives the separation of the two daughter cells. A key molecule in this process is the inositol lipid PtdIns(4,5)P_2_, which recruits numerous factors to the equatorial region for contractile ring assembly. Despite the importance of PtdIns(4,5)P_2_ in cytokinesis, the regulation of this lipid in cell division remains poorly understood. Here, we identify a role for IPIP27 in mediating cellular PtdIns(4,5)P_2_ homeostasis. IPIP27 scaffolds the inositol phosphatase oculocerebrorenal syndrome of Lowe (OCRL) by coupling it to endocytic BAR domain proteins. Loss of IPIP27 causes accumulation of PtdIns(4,5)P_2_ on aberrant endomembrane vacuoles, mislocalization of the cytokinetic machinery, and extensive cortical membrane blebbing. This phenotype is observed in *Drosophila* and human cells and can result in cytokinesis failure. We have therefore identified IPIP27 as a key modulator of cellular PtdIns(4,5)P_2_ homeostasis required for normal cytokinesis. The results indicate that scaffolding of inositol phosphatase activity is critical for maintaining PtdIns(4,5)P_2_ homeostasis and highlight a critical role for this process in cell division.

## Highlights

• IPIP27 scaffolds the inositol phosphatase OCRL via coupling to BAR domain proteins
• IPIP27 scaffolding of OCRL is critical for cellular PtdIns(4,5)P_2_ homeostasis
• IPIP27 is required for cortical actin and membrane stability during cytokinesis
• IPIP27 function is conserved from flies to humans

## Graphical Abstract

**Figure:**
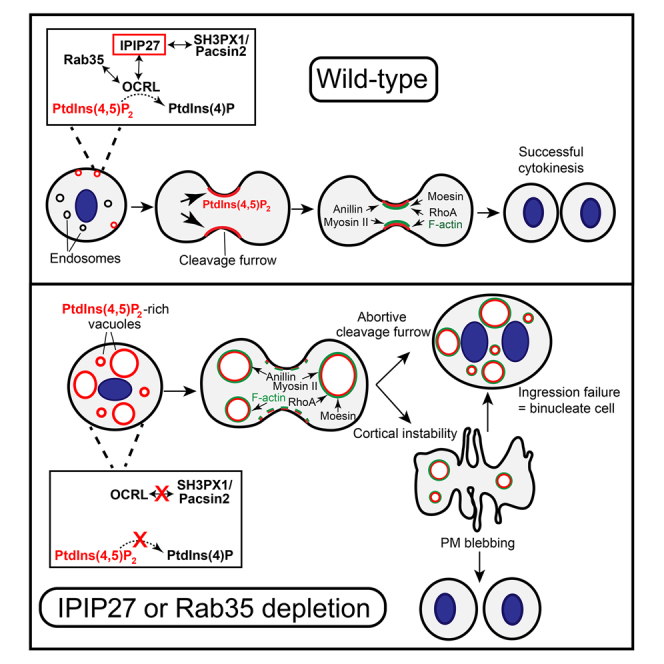

## Teaser

Carim et al. reveal that the IPIP27 protein, which physically couples the inositol phosphatase OCRL to endocytic BAR domain proteins, is required for cellular phosphoinositide homeostasis and normal cell division. The results show the importance of physically scaffolding inositol phosphatase activity within cells.

## Introduction

Cytokinesis, the final step of cell division, is a fundamental process that is required for organismal development and tissue homeostasis. During cytokinesis, an actin-based contractile ring forms in the equatorial region of the dividing mother cell and subsequently constricts to physically divide the cytoplasm [1, 2, 3]. Cytokinesis is completed by the process of abscission, resulting in complete physical separation of the two daughter cells [4]. The contractile ring comprises primarily actin, myosin II, and formins, with various associated factors that include the scaffolding protein anillin [5]. The primary driver of contractile ring assembly is RhoA, which becomes activated by the Ect2 Rho-GEF in the equatorial region of the plasma membrane [1, 2, 3, 6]. Another key player is the phosphoinositide lipid PtdIns(4,5)P_2_, which is also enriched in the equatorial region [7, 8, 9]. PtdIns(4,5)P_2_ is a strong inducer of actin assembly [10], and within the equatorial region it promotes the recruitment of various factors including Ect2 and anillin [11, 12], as well as ezrin, radixin, and moesin (ERM) proteins that help link the actin cytoskeleton to the plasma membrane [13]. At a late stage of cytokinesis, PtdIns(4,5)P_2_ is removed to promote actin disassembly, which is a prerequisite for membrane abscission [14]. It is therefore important that the synthesis and removal of PtdIns(4,5)P_2_ are tightly controlled to ensure effective cytokinesis [9].

Mammalian oculocerebrorenal syndrome of Lowe (OCRL1) is an inositol 5-phosphatase whose preferred substrate is PtdIns(4,5)P_2_ [15, 16]. Mutation of OCRL1 in humans causes Lowe syndrome [17] and Dent-2 disease [18], which result in neurological, ocular, and renal defects [19, 20]. OCRL1 is localized to various endomembrane compartments including the *trans*-Golgi [21, 22], endosomes [22, 23, 24, 25], and lysosomes [26]. OCRL1 has been implicated in a number of cellular functions including endocytic traffic (reviewed in [27]), where recent studies have shown it can hydrolyze PtdIns(4,5)P_2_ during the uncoating or maturation of clathrin-coated vesicles [28, 29]. In cytokinesis of mammalian cells, OCRL1 is recruited to the intercellular bridge by Rab35, where it hydrolyzes PtdIns(4,5)P_2_ to promote actin dissolution and abscission [14]. In contrast to mammals, which also express the OCRL1 paralog INPP5B that may partially compensate for loss of OCRL1 [30], *Drosophila* have only a single enzyme, *Drosophila* ortholog of OCRL (dOCRL) [31, 32]. Depletion of dOCRL from cultured *Drosophila* cells results in a more dramatic cytokinesis phenotype, with failure at the ingression stage, resulting in binucleation [31]. In dOCRL-depleted cells, PtdIns(4,5)P_2_ accumulates on internal vacuoles, which in turn causes aberrant recruitment of the cytokinetic machinery to these compartments, its depletion from the cortex, and ingression failure [31]. dOCRL is therefore required to maintain cellular PtdIns(4,5)P_2_ homeostasis, which is important for cell division. The lack of genetic redundancy in *Drosophila* likely explains the severity of the cytokinesis phenotype in this organism compared to mammals.

Although dOCRL and OCRL1 have been identified as important regulators of PtdIns(4,5)P_2_ during cytokinesis, the mechanisms by which they function in this process remain poorly understood. In this study, we investigated the OCRL1 binding partner IPIP27 (inositol phosphatase interacting protein of 27 kDa), which exists as two paralogs in humans, IPIP27A and IPIP27B (also known as Ses1 and Ses2) [33, 34]. IPIP27A and B both function in endocytic traffic [34]. IPIP27A acts as a scaffold protein, physically linking OCRL1 to the actin-associated protein pacsin 2 [35], which is able to recognize or induce membrane curvature via its amino-terminal F-BAR domain [36, 37]. The ability of IPIP27A to link OCRL1 and pacsin 2 is important for biogenesis of trafficking intermediates, likely by concentrating OCRL1 at sites of carrier formation [35]. Here, using both *Drosophila* and mammalian cells, we identify the importance of IPIP27 in maintaining cellular PtdIns(4,5)P_2_ homeostasis, which in turn is important for cortical actin and plasma membrane integrity during cleavage furrow ingression. IPIP27 function is dependent upon binding to both OCRL and BAR domain proteins, indicating that its ability to scaffold OCRL activity is critical to maintain correct cellular PtdIns(4,5)P_2_ distribution. The results provide new insight into how PtdIns(4,5)P_2_ homeostasis is controlled within cells and highlight the importance of this process for cell division.

## Results

### Conservation of IPIP27 in *Drosophila*

**Figure 1:**
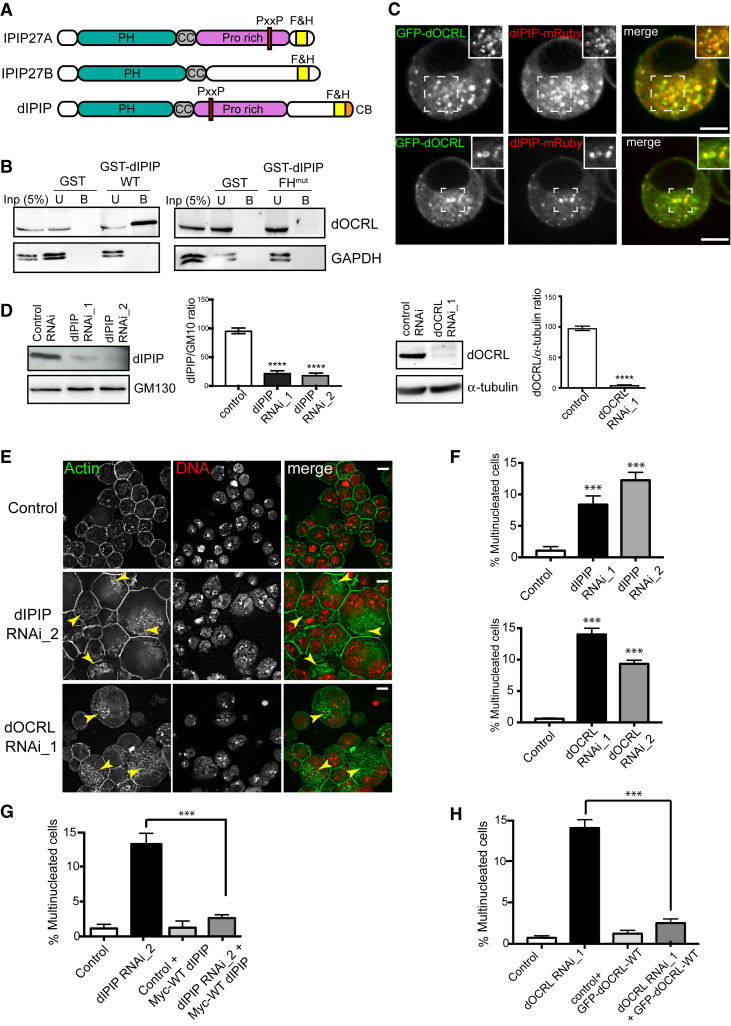
dIPIP Interacts with dOCRL and Is Required for Cytokinesis (A) Schematic of human IPIP27A and IPIP27B and *Drosophila* dIPIP. (B) Pull-down using GST- dIPIP wild-type (WT) or F&H mutant (F267A) and *Drosophila* S2 cell lysate. Input (5%), unbound (5%), and bound fractions (50%) were blotted. (C) Confocal microscopy of dIPIP-mRuby (red) co-expressed with GFP-dOCRL (green) in live S2 cells. Scale bar, 5 μm. (D) Western blot showing RNAi-mediated depletion of dIPIP (left) or dOCRL (right) in S2 cells. Bar graphs show relative protein abundance. Values are means ± SEM of 3 independent experiments each done in triplicate, ^∗∗∗∗^p < 0.0001, Student’s t test. (E) RNAi-treated S2 cells were stained with Alexa 488-phalloidin (green, F-actin) and Hoechst 33342 (red, DNA). Arrowheads point to cytoplasmic actin-positive vacuoles. Scale bar, 5 μm. (F) Quantitation of multinucleation. Bars represent the mean ± SEM of 4 experiments with ∼500 cells per experiment. ^∗∗∗^p < 0.0001, Student’s t test. (G and H) Rescue of multinucleation by wild-type Myc-dIPIP (G) or GFP-dOCRL (H). Bars represent the mean ± SEM of 4 experiments with ∼500 cells per experiment. ^∗∗∗^p < 0.0001, Student’s t test. See also Figure S1.

IPIP27 exists as two paralogs in mammals, named IPIP27A and IPIP27B (Ses1 and Ses2) [33, 34], whereas only a single ortholog is present in *Drosophila melanogaster*, which we refer to as dIPIP (Figure 1A). As expected from the mammalian proteins [33, 34], dIPIP interacts with the single OCRL and INPP5B ortholog in *Drosophila*, dOCRL, through its F&H motif (Figure 1B). The C-terminal region of dIPIP also contains a conserved PxxP motif (^151^PxPPPRR^157^) that in mammalian IPIP27A binds the SH3 domain of pacsin 2 [35], and a putative clathrin-binding site at its C terminus that is not present in mammalian IPIP27. Localization experiments in *Drosophila* S2 cells indicated the presence of dIPIP on cytoplasmic puncta that likely correspond to endocytic compartments, where it colocalizes with dOCRL (Figure 1C; see also Figure 7C) [32]. IPIP27 interaction and co-localization with OCRL is therefore conserved in *Drosophila melanogaster*.

### Depletion of dIPIP Results in Cytokinesis Failure

Given that depletion of dOCRL results in a penetrant cytokinesis defect [31], we explored whether loss of dIPIP may also disrupt cytokinesis. dIPIP was depleted from S2 cells using double-stranded RNA (dsRNA) (Figure 1D), and cytokinesis failure assessed by counting the degree of multi-nucleation. As shown in Figures 1E and 1F, there was significant cytokinesis failure upon dIPIP depletion. This effect was observed with two distinct dsRNAs and could be rescued by re-expression of dIPIP in the depleted cells (Figure 1G), confirming specificity. The extent of multi-nucleation was comparable to that seen upon dOCRL depletion (Figures 1E and 1F), which was also rescued upon re-expression of dOCRL (Figures 1F and 1H). Interestingly, depletion of dOCRL led to a loss of dIPIP protein (Figure S1A), and a similar phenomenon is observed in mammalian cells depleted of OCRL1 (Figure S1B), suggesting that binding to OCRL stabilizes IPIP27. In contrast, IPIP27 does not affect OCRL levels in *Drosophila* (Figure S1A) or mammalian cells (Figure S1B).

### Dysregulation of PtdIns(4,5)P_2_ Homeostasis upon dIPIP Depletion

**Figure 2:**
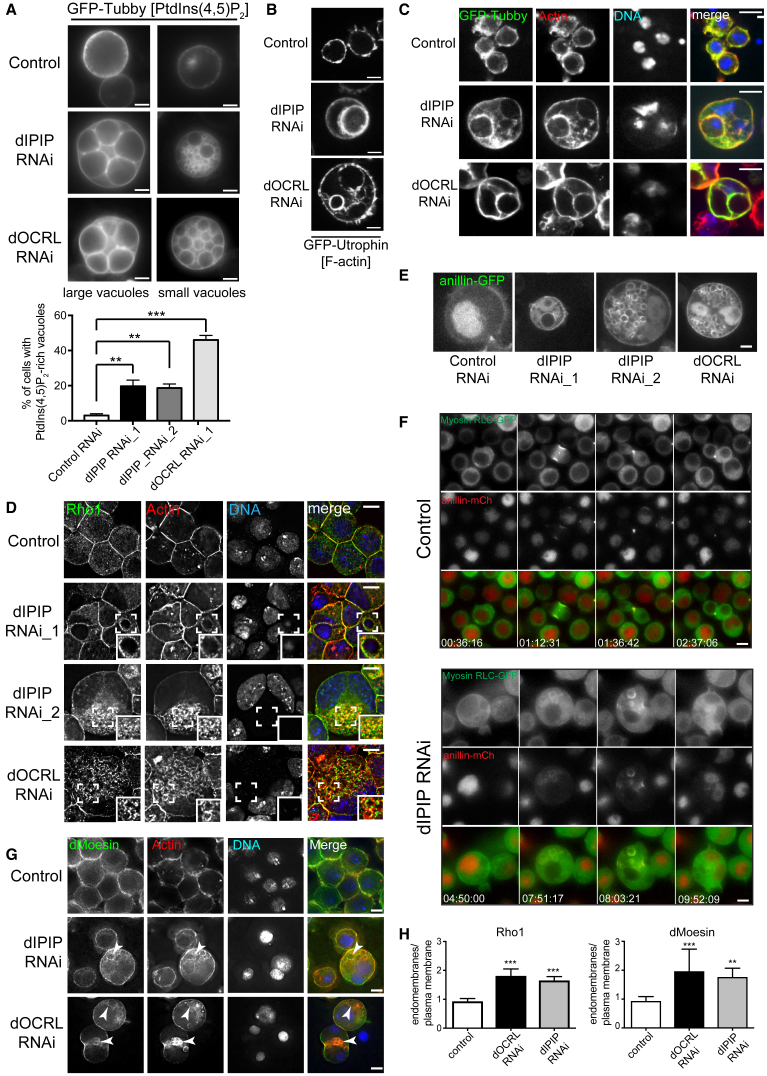
dIPIP Depletion Gives Rise to PtdIns(4,5)P_2_- and Actin-Rich Vacuoles with Mislocalized Cytokinetic Machinery (A) Top: live stills of control, dIPIP, or dOCRL-depleted S2 cells stably expressing GFP-Tubby. Scale bar, 5 μm. Bottom: quantitation of PtdIns(4,5)_2_-rich vacuoles. Bars represent the mean of 3 experiments with >100 cells per condition per experiment. Error bars indicate SEM and ^∗∗^p < 0.005, ^∗∗∗^p < 0.0001, Student’s t test. (B) Live stills of RNAi-treated S2 cells stably expressing GFP-Utrophin (actin). Scale bar, 5 μm. (C) RNAi-treated S2 cells stably expressing GFP-Tubby (green), fixed and labeled with phalloidin (F-actin, red) and Hoechst 33342 (DNA, blue). Scale bar, 5 μm. (D) RNAi-treated S2 cells labeled with anti-Rho1 (green), phalloidin-TRITC (red), and Hoechst 33342 (blue). Scale bar, 5 μm. (E) Live stills of RNAi-treated S2 cells stably expressing anillin-GFP. (F) Selected time-lapse frames of dividing control or dIPIP-depleted cells stably expressing anillin-mCherry (red) and Spaghetti-squash (*Drosophila* Myosin RLC)-GFP (green). Times are in hours, minutes, and seconds from start of imaging. (G) RNAi-treated S2 cells labeled with anti-dMoesin (green), phalloidin Alexa 594 (red), and Hoechst 33342 (blue). Arrowheads indicate cytoplasmic vacuoles. (H) Quantitation of ratio of Rho1 and dMoesin at endomembranes versus plasma membrane. Bars indicate mean ± SD from 3 independent experiments. ^∗∗^p < 0.005, ^∗∗∗^p < 0.0002, one-sample t test. Scale bars, 5 μm. See also Figure S2 and Videos S1 and S2.

To investigate the molecular basis of the cytokinesis defect seen upon dIPIP depletion, which we hypothesized was due to altered PtdIns(4,5)P_2_ homeostasis, dIPIP was depleted from S2 cells stably expressing GFP-Tubby as a PtdIns(4,5)P_2_ biosensor [38]. Strikingly, dIPIP-depleted cells showed a dramatic accumulation of PtdIns(4,5)P_2_ on intracellular vacuoles, and a lack of PtdIns(4,5)P_2_ enrichment at the cleavage furrow during division (Figure 2A; Video S1). PtdIns(4,5)P_2_ accumulation on intracellular vacuoles was also observed in dOCRL-depleted cells (Figure 2A), as seen previously [31]. The results are therefore consistent with dIPIP and dOCRL acting together to maintain PtdIns(4,5)P_2_ homeostasis. PtdIns(4,5)P_2_ is a strong promoter of actin assembly, and we detected strong actin enrichment on the vacuolar membranes (Figure 2B), where it colocalized with GFP-Tubby (Figure 2C). Although depletion of dIPIP or dOCRL caused enlargement of endosomal compartments, consistent with a role for these proteins in the endocytic pathway (Figures S2A–S2C) [31, 32], the larger vacuoles are largely devoid of endosome markers (Figures S2D and S2E). We believe it most likely that the vacuoles derive from endosomes, but, due to their altered phosphoinositide composition, with ectopically accumulated PtdIns(4,5)P_2_, they have lost their endosomal identity [39].

Video S1. Mislocalization of PtdIns(4,5)P_2_ in Dividing S2 Cells upon dIPIP Depletion, Related to Figure 2 Control or dIPIP-depleted S2 cells stably expressing GFP-Tubby (green) and tubulin-mCherry (red) were imaged using time-lapse confocal fluorescence microscopy. Frames were collected every min over 2.5 h and the video is displayed at 3 frames/second.

### Mislocalization of Cytokinetic Machinery upon dIPIP Depletion

The accumulation of PtdIns(4,5)P_2_ on intracellular vacuoles raised the possibility that cytokinetic machinery normally targeted to the cleavage furrow through binding PtdIns(4,5)P_2_ [9] may be mis-targeted to the vacuoles. Indeed, we observed accumulation of Rho1, or stably expressed anillin-GFP, on the internal actin-rich vacuoles (Figures 2D and 2E). Live imaging also showed accumulation of GFP-tagged myosin II regulatory light chain (MRLC) and anillin-mCherry on the vacuoles of dIPIP-depleted cells undergoing division, indicated by the presence of anillin outside the nucleus [40], whereas in control cells undergoing cytokinesis, they localized to the contractile ring, as expected (Figure 2F; Video S2). We also observed vacuolar accumulation of dMoesin, the single ERM protein in *Drosophila* involved in linking cortical actin to PtdIns(4,5)P_2_ [13, 41] (Figure 2G). Quantitation revealed a depletion of Rho1 and dMoesin from the plasma membrane, and a reciprocal enrichment on endomembranes (Figure 2H). The mis-localization of key cytokinetic machinery to the PtdIns(4,5)P_2_- and actin-rich vacuoles is similar to that seen upon dOCRL depletion [31](Figures 2D, 2E, and 2G) and may explain the cytokinesis failure seen in dIPIP-depleted cells.

Video S2. Mislocalization of Myosin Regulatory Light-Chain GFP and Anillin-mCherry to Internal Vacuoles in Dividing S2 Cells upon dIPIP Depletion, Related to Figure 2 Control or dIPIP-depleted S2 cells stably expressing anillin-mCherry (red) and myosin regulatory light chain (MRLC)-GFP (green) imaged using time-lapse fluorescence microscopy. Frames were collected every 12 min over 16 h and the video is displayed at 4 frames/second.

### Depletion of dIPIP Causes Cortical Actin Instability

**Figure 3:**
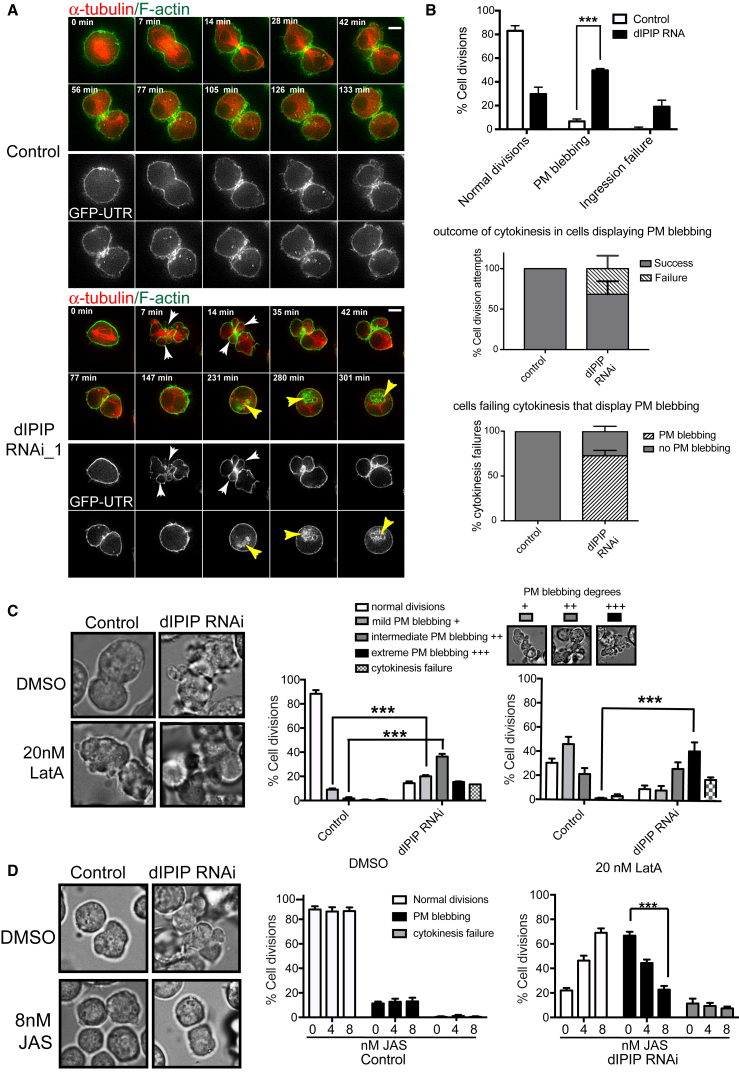
Depletion of dIPIP Causes Cortical Actin Instability during Cytokinesis (A) Live stills showing dynamics of GFP-Utrophin in control or dIPIP-depleted cells during cell division. White arrowheads show cortical blebs and yellow arrowheads mark intracellular vacuoles. Time is in minutes from the last frame of metaphase. Scale bar, 5 μm. (B) Top: quantification of cell division phenotypes. Middle, quantification of cell division outcome in blebbing cells. Bottom: quantitation of cells failing cytokinesis that had undergone blebbing. Bars represent mean from 3 experiments with 80–100 cells per experiment. Error bars show SEM. ^∗∗∗^p < 0.0005, chi-square analysis. (C and D) Left: bright-field stills of control or dIPIP-depleted cells treated with DMSO, 20 nM latrunculin A (LatA; C), or 8 nM jasplakinolide (JAS; D). Right: quantification of phenotypes upon LatA (C) or JAS (D) treatment. Bars represent the mean of 3 experiments with >100 cells analyzed per condition per experiment. Error bars represent SEM. ^∗∗∗^p < 0.0005, chi-square analysis. See also Video S3.

To better understand the cytokinetic defect in dIPIP depletion, time-lapse imaging of dividing cells was performed. Analysis of cells stably expressing GFP-utrophin to label F-actin in the cell cortex, and α-tubulin-mCherry to label microtubules, revealed a striking perturbation of cortical membrane dynamics, with extensive membrane blebbing (Figure 3A; Video S3). The blebbing occurred post-anaphase, mainly in the equatorial region, and was distinct from the natural blebbing of the polar cortex normally seen in cell division [13, 42, 43, 44]. The blebbing phenotype was penetrant, with ∼50% of the cells displaying blebbing in the equatorial region (Figure 3B, top), of which ∼30% underwent cytokinesis failure, with the remainder successfully dividing (Figure 3B, middle). Of the cells undergoing cytokinesis failure, ∼70% had previously undergone blebbing, indicating that some cells fail cytokinesis without blebbing (Figure 3B, bottom). This may reflect a more severe ingression defect in these cells preventing the formation of a cleavage furrow where blebbing is normally observed. The extensive blebbing upon dIPIP depletion suggested a defect in cortical actin, most likely reduced stability within the equatorial region. To test this, cells were treated with the actin destabilizing drug latrunculin A [45] and blebbing assessed. As shown in Figure 3C, latrunculin A induced blebbing of control cells, but the extent of blebbing was greatly increased upon dIPIP depletion, indicating sensitization to the drug. In contrast, treatment with the actin stabilizing drug jasplakinolide [46] efficiently rescued the blebbing of dIPIP-depleted cells, with no effect on control cells (Figure 3D). These results indicate that depletion of dIPIP causes aberrant blebbing of the equatorial plasma membrane, which is a consequence of reduced cortical actin stability.

Video S3. Cortical Membrane Blebbing and Cytokinesis Failure in dIPIP-Depleted S2 Cells, Related to Figure 3 Control or dIPIP-depleted S2 cells stably expressing GFP-utrophin (green) and tubulin-mCherry (red) imaged using time-lapse fluorescence microscopy. Frames were collected every 7 min over 16 h and the video is displayed at 4 frames/second.

### Cortical Actin Instability upon Depletion of Mammalian IPIP27A

**Figure 4:**
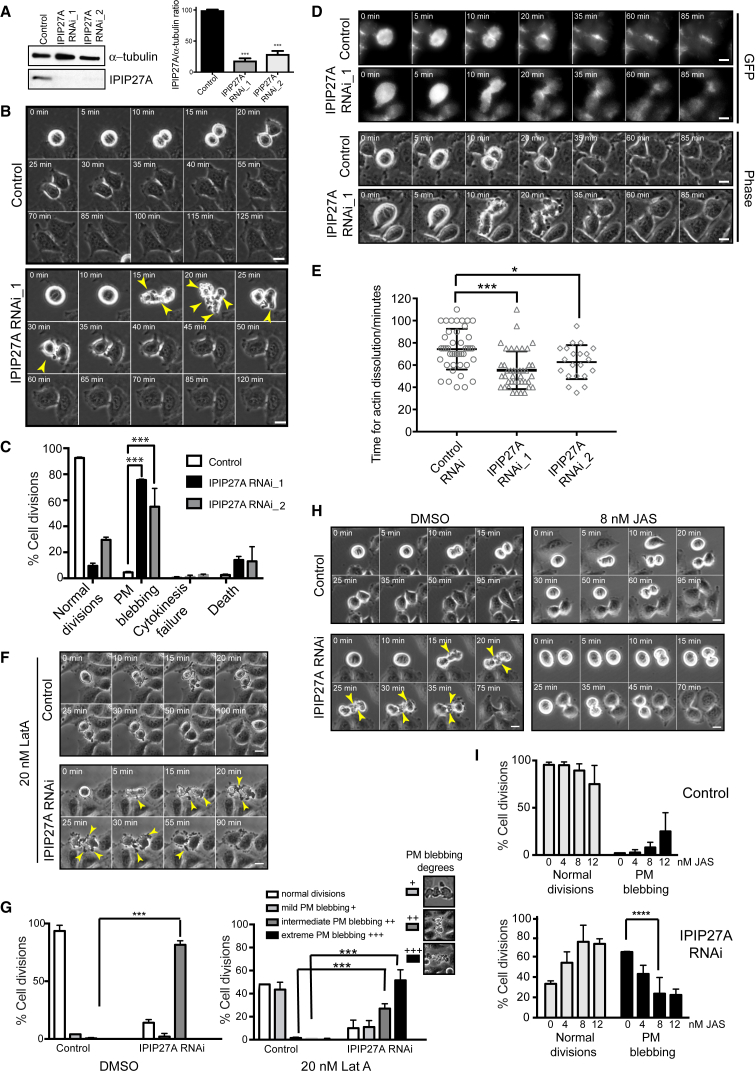
Depletion of Human IPIP27A Causes Cortical Actin Instability during Cytokinesis (A) Western blot showing IPIP27A depletion using two independent siRNAs. Bar graph shows relative protein abundance. Values are means ± SEM of 3 independent experiments, each done in triplicate, ^∗∗∗^p < 0.0001, Student’s t test. (B) Time-lapse bright-field still images of control or IPIP27A-depleted cells during cell division. Arrowheads indicate plasma membrane blebs. Time is in minutes from metaphase. (C) Quantification of phenotypes. Bars represent the mean of 3 experiments with 100–150 cells analyzed per condition per experiment. Error bars represent SEM. ^∗∗∗^p < 0.0001, chi-square analysis. (D) Fluorescence and phase contrast time-lapse live stills of control versus IPIP27A-depleted HeLa cells stably expressing Lifeact-GFP. (E) Scatterplot of the time for dissolution of Lifeact-GFP from the contractile ring. Bars represent the mean with SD. ^∗^p < 0.05 ^∗∗∗^p < 0.0001, Mann-Whitney test (F and H) Time-lapse bright-field stills of control and IPIP27A-depleted cells treated with DMSO, 20 nM LatA (F), or 8 nM JAS (H). Arrowheads indicate plasma membrane blebs. (G and I) Quantification of phenotypes after treatment with LatA (G) or JAS (I). Bars represent the mean of 3 experiments with 100–150 cells analyzed per condition per experiment. Error bars indicate SEM. ^∗∗∗^p < 0.0005, chi-square analysis. Scale bars, 10 μm. See also Figure S1 and Videos S4 and S5.

To assess whether IPIP27 function is conserved in mammals, IPIP27A and IPIP27B were depleted from HeLa cells (Figures 4A and S1C) and cell division followed by live imaging. IPIP27A depletion resulted in dramatic post-anaphase blebbing of the plasma membrane in the equatorial region (Figure 4B; Video S4), similar to that seen in dIPIP-depleted S2 cells. In contrast, no blebbing was observed upon IPIP27B depletion (Figures S1D and S1E). Blebbing was observed with two independent siRNAs to IPIP27A, confirming specificity of the phenotype (Figure 4C). To determine whether the blebbing was due to dysregulation of cortical actin, actin was visualized in dividing cells using LifeAct. In control cells, actin persisted in the equatorial region until late cytokinesis, at which point staining was lost during actin filament dissolution (Figure 4D). Upon IPIP27A depletion, the equatorial pool of actin appeared to dissolve earlier than in control cells, suggesting instability of the cortical actin in this region (Figures 4D and 4E). This is further supported by the observed sensitization of IPIP27A-depleted cells to latrunculin A, (Figures 4F and 4G), and the rescue of blebbing with low doses of jasplakinolide (Figures 4H and 4I; Video S5). These results indicate that depletion of mammalian IPIP27A results in extensive plasma membrane blebbing at the equatorial region during cytokinesis, which is due to reduced cortical actin stability in this region.

Video S4. IPIP27A Depletion Causes Extensive Plasma Membrane Blebbing in Dividing HeLa Cells, Related to Figure 4 Control or IPIP27A-depleted HeLa cells imaged using time-lapse phase contrast microscopy. Frames were collected every 5 min over 16 h and the video is displayed at 3 frames/second.

Video S5. Rescue of IPIP27A Depletion-Induced Plasma Membrane Blebbing by the Actin Stabilizing Drug Jasplakinolide, Related to Figure 4 Control or IPIP27A-depleted HeLa cells were treated with 8 nM jasplakinolide and imaged using time-lapse phase contrast microscopy. Frames were collected every 5 min over 16 h and the video is displayed at 4 frames/second.

### Cortical Actin Instability upon dIPIP or IPIP27A Depletion Is Due to Dysregulation of PtdIns(4,5)P_2_

**Figure 5:**
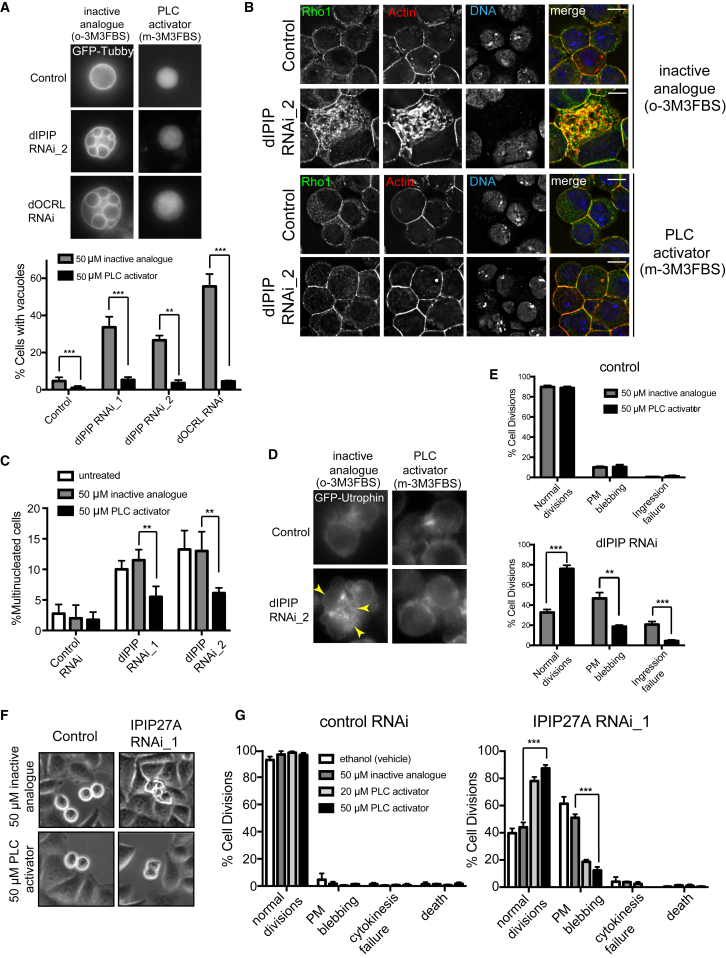
Cortical Instability and Cytokinesis Failure upon dIPIP or IPIP27A Depletion Is Due to Dysregulated PtdIns(4,5)P_2_ Homeostasis (A) Top: live stills of S2 cells stably expressing GFP-Tubby depleted of dIPIP or dOCRL and treated with 50 μM inactive analog (*o*-3M3FBS) or PLC activator (*m*-3M3FBS). Bottom: quantification of PtdIns(4,5)P_2_-rich vacuoles. Bars represent the mean of 3 experiments with 250–400 cells analyzed per condition per experiment. ^∗∗^p < 0.001, ^∗∗∗^p < 0.0001, Student’s t test. (B) S2 cells depleted of dIPIP or dOCRL and treated with 50 μM of inactive analog or PLC activator, followed by staining with anti-Rho1 (green), phalloidin-TRITC (red), and Hoechst 33342 (blue). Scale bar, 5 μm. (C) Quantitation of multinucleation in S2 cells depleted of dIPIP and treated with 50 μM inactive analog or PLC activator. Bars represent the mean of 3 experiments with 350–500 cells analyzed per condition per experiment. ^∗∗∗^p < 0.0001, ^∗∗^p < 0.001, ^∗^p < 0.05, Student’s t test. (D) Live time-lapse stills of control or dIPIP-depleted S2 cells stably expressing GFP-Utrophin treated with inactive analog or PLC activator. Arrowheads indicate plasma membrane blebs during cytokinesis. (E) Quantification of cell division phenotypes, showing the mean of 3 experiments with 50 cells analyzed per condition per experiment. Error bars indicate SEM. ^∗∗∗^p < 0.0001, ^∗∗^p < 0.001, chi-square analysis. (F) Live time-lapse stills of control or IPIP27A-depleted HeLa cells following treatment with 50 μM inactive analog or PLC activator. (G) Quantification of phenotypes, showing the mean of 3 experiments with 150–200 cells analyzed per condition per experiment. Error bars indicate SEM. ^∗∗∗^p < 0.0001, chi-square analysis. See also Figure S3.

We reasoned that dysregulation of PtdIns(4,5)P_2_ may underlie the cortical instability seen upon loss of dIPIP in *Drosophila* cells and IPIP27A in human cells. In S2 cells, PtdIns(4,5)P_2_ accumulation on intracellular vacuoles causes mis-targeting of the actin-associated cytokinetic machinery to these vacuoles and its depletion from the cortex (Figure 2). To more directly test PtdIns(4,5)P_2_ involvement in the cytokinetic phenotypes, S2 cells were treated with the phospholipase C (PLC) activator *m*-3M3FBS, which rescues the PtdIns(4,5)P_2_ vacuolar phenotype seen upon dOCRL depletion (K.B.E.K. and S.C., unpublished data). As shown in Figures 5A–5E, treatment of dIPIP-depleted S2 cells with *m*-3M3FBS, but not the inactive analog *o*-3M3FBS, rescued the PtdIns(4,5)P_2_ vacuoles, the mis-targeting of cytokinetic machinery to the vacuoles, cortical membrane blebbing, and cytokinetic failure, confirming that these phenotypes all derive from PtdIns(4,5)P_2_ dysregulation. Although we did not detect PtdIns(4,5)P_2_-positive vacuoles in IPIP27A-depleted human cells, the similarity in cortical phenotype with dIPIP-depleted S2 cells suggested a similar underlying mechanism. This was confirmed by rescue of cortical membrane blebbing in IPIP27A-depleted cells by *m*-3M3FBS (Figures 5F and 5G).

Depletion of dOCRL from S2 cells also caused cortical membrane blebbing, although it was less extensive than that observed upon dIPIP depletion (Figures S3A and S3B). In contrast, depletion of OCRL1 or INPP5B from HeLa cells, alone or together, did not cause blebbing (Figure S3C). This may be due to redundancy with other mammalian inositol 5-phosphatases, of which there are many [47]. Nevertheless, the fact that depletion of dOCRL in *Drosophila* S2 cells phenocopies loss of dIPIP supports the idea that dIPIP acts through dOCRL to control PtdIns(4,5)P_2_ homeostasis for cortical membrane integrity during cytokinesis. In mammalian cells the blebbing phenotype is also dependent upon PtdIns(4,5)P_2_ dysregulation but may involve other 5-phosphatases in addition to OCRL1 or INPP5B.

### dIPIP Interactions Are Important for Successful Cytokinesis

**Figure 6:**
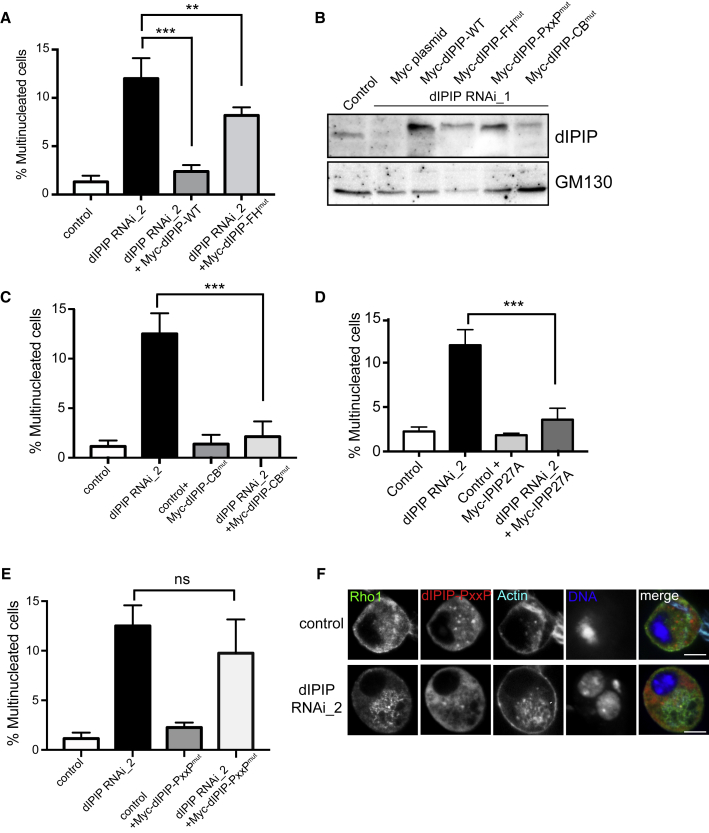
dIPIP Binding to dOCRL and SH3PX1 Is Required for Successful Cytokinesis (A) Quantification of multinucleation upon expression of Myc-tagged wild-type (WT) dIPIP or the dOCRL binding-deficient F&H mutant (F267A) in dIPIP-depleted S2 cells. ^∗∗∗^p < 0.0001, ^∗∗^p < 0.001, Student’s t test. (B) Western blot showing dIPIP depletion and expression of Myc-tagged rescue constructs. (C and D) Quantification of multinucleation upon expression of Myc-tagged clathrin binding-deficient mutant (CB^mut^, ^293^LIQL^296^ > AAAA) dIPIP (C) or human IPIP27A (D) in dIPIP-depleted S2 cells. ^∗∗∗^p < 0.0001. (E) Quantitation of multinucleation upon expression of Myc-tagged dIPIP PxxP mutant in dIPIP-depleted S2 cells. ^∗∗∗^p < 0.0001. In (A), and (C)–(E), bars represent the mean of 3 experiments with 200–250 cells analyzed per condition per experiment. Error bars indicate SEM, and p values are from a Student’s t test. (F) Control or dIPIP-depleted S2 cells expressing dIPIP PxxP mutant were fixed and labeled with anti-Rho1 (green), phalloidin-Alexa 647 (far red), and Hoechst 33342 (blue). Arrowheads point to cytoplasmic vacuoles. Scale bar, 5 μm. See also Figure S4.

dIPIP contains several protein-protein interaction motifs (Figure 1A). To determine the functional importance of these motifs, rescue experiments were performed. In contrast to wild-type dIPIP, the F&H mutant, which is unable to bind dOCRL (Figure 1B), resulted in a severely reduced ability to rescue the multi-nucleation phenotype (Figures 6A and 6B). Mutation of the predicted clathrin box in dIPIP had no effect upon rescue, indicating that dIPIP binding to clathrin is dispensable for cytokinesis (Figure 6C). Human IPIP27A was also able to rescue dIPIP depletion, confirming functional conservation of IPIP27 between species (Figure 6D). Both IPIP27A and dIPIP have a conserved PxxP motif, which in IPIP27A is able to bind to SH3 domain proteins such as pacsin 2 [35]. As shown in Figures 6E and 6F, mutation of the PxxP motif in dIPIP strongly impaired rescue of actin-rich vacuole formation, mis-targeting of Rho1 to these vacuoles, as well as multi-nucleation, indicating the functional importance of this motif, and therefore binding of dIPIP to an SH3 domain partner.

### dIPIP Binds SH3PX1, which Is Important for Cortical Membrane Stability in Cytokinesis

**Figure 7:**
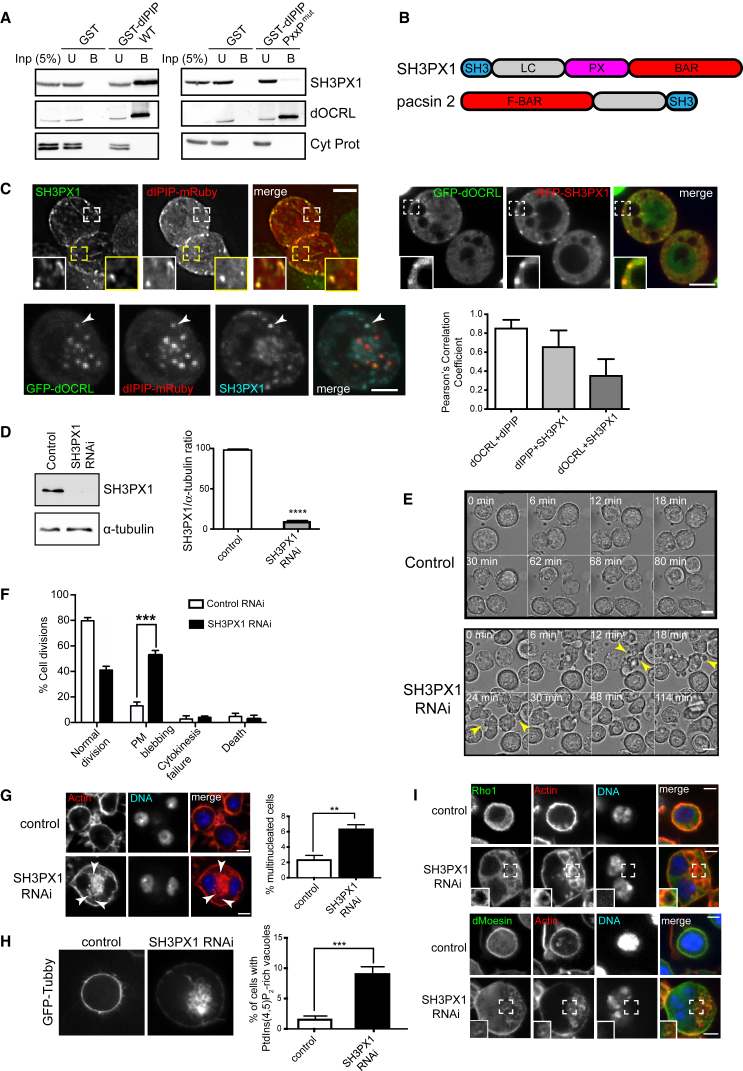
Depletion of SH3PX1 or Pacsin 2 Phenocopies IPIP Depletion in *Drosophila* or Human Cells, Respectively (A) Pull-down assay using GST-dIPIP or GST-dIPIP PxxP mutant (^156^RR^157^ > AA) and *Drosophila* S2 cell lysate, followed by western blotting of input (5%), unbound (5%), and bound fractions (50%). (B) Schematic of SH3PX1 and pacsin 2. (C) Top left: S2 cells expressing dIPIP-mRuby were fixed and labeled for SH3PX1. Top right: live still of S2 cells expressing GFP-dOCRL and RFP-SH3PX1. Bottom left: S2 cells transiently expressing GFP-dOCRL and dIPIP-mRuby and fixed and labeled for SH3PX1. Arrowheads indicate colocalization in puncta. Bottom right: colocalization analysis using Pearson’s correlation coefficient. Bars represent means ± SEM from 3 experiments with ∼30 cells per experiment Scale bar, 5 μm. (D) Western blot showing SH3PX1 depletion. The bar graph shows relative protein abundance. Values are means ± SEM of 3 independent experiments each done in triplicate. ^∗∗∗∗^p < 0.0001, Student’s t test. (E) Phase contrast time-lapse stills of control or SH3PX1-depleted cells undergoing cell division. Time is in minutes from metaphase. Scale bar, 5 μm. (F) Quantification of phenotypes. Bars represent the mean of 3 experiments with ∼60 cells analyzed per condition per experiment. Error bars represent SEM. ^∗∗∗^p < 0.0001, chi-square analysis. (G) Left: RNAi-treated S2 cells were stained with phalloidin (red, F-actin) and Hoechst 33342 (blue, DNA). Arrowheads indicate actin-positive vacuoles. Right: quantitation of multinucleation. Bars represent the mean ± SEM of 3 experiments with ∼500 cells per experiment. ^∗∗∗^p < 0.0001, Student’s t test. (H) Left: live stills of control or SH3PX1-depleted S2 cells stably expressing GFP-Tubby. Right: quantitation of PtdIns(4,5)_2_-rich vacuoles. Bars represent the mean ± SEM of 3 experiments with ∼300 cells per condition per experiment. ^∗∗∗^p < 0.0001, Student’s t test. (I) RNAi-treated S2 cells labeled with anti-Rho1 anti-dMoesin (green), phalloidin (red), and Hoechst 33342 (blue). Scale bars, 5 μm. See also Figures S4–S6 and Video S6.

To identify the relevant binding partner for the dIPIP PxxP motif, we first analyzed syndapin, the *Drosophila* ortholog of pacsin 2, but it failed to interact with dIPIP (Figure S4A). Instead, we observed strong binding of dIPIP to SH3PX1, the *Drosophila* ortholog of SNX9 [48], in agreement with a previous genome-wide interactome study [49], and binding was dependent upon the PxxP motif (Figure 7A). In addition to the amino-terminal SH3 domain, SH3PX1 contains a lipid binding PX domain and a BAR domain that is likely involved in membrane curvature sensing or generation (Figure 7B). SH3PX1 was localized to the cell cortex but could also be detected on puncta that likely correspond to endocytic structures [50], where there was colocalization with dIPIP and dOCRL (Figure 7C). Interestingly, labeling for SH3PX1 in dIPIP- or dOCRL-depleted cells indicated a striking accumulation on internal actin-positive compartments, which may reflect its association with this actin pool (Figure S4B). We next wanted to determine the requirement for SH3PX1 for successful cytokinesis. Depletion of SH3PX1 from S2 cells (Figure 7D) resulted in similar phenotypes to those seen upon depletion of dIPIP or dOCRL, with extensive plasma membrane blebbing (Figures 7E and 7F; Video S6), increased multi-nucleation (Figure 7G), and accumulation of actin-rich vacuoles that also contained PtdIns(4,5)P_2_, Rho1, and dMoesin (Figures 7G, 7H, and 7I). Together, the results are consistent with a functional interaction between dIPIP and SH3PX1 that is required for PtdIns(4,5)P_2_ homeostasis and cortical membrane stability during cytokinesis.

Video S6. Cortical Membrane Blebbing in Dividing S2 Cells upon SH3PX1 Depletion, Related to Figure 7 Control or SH3PX1-depleted S2 cells were imaged using time-lapse fluorescence microscopy. Frames were collected every 6 min over 13 h and the video is displayed at 4 frames/second.

### Pacsin 2 Supports Cortical Actin Stability in Mammalian Cell Cytokinesis

To determine the requirement for the IPIP27A partner pacsin 2 (Figure S4C) for successful cytokinesis in mammalian cells, it was depleted from HeLa cells and cytokinesis visualized by live imaging. Pacsin 2 depletion (Figure S4C) gave a penetrant membrane blebbing phenotype (Figures S4D and S4E), whereas depletion of the other described IPIP27A binding partners, CD2AP or myosin 1E [35], did not (Figures S5A and S5B). Depletion of SNX9 also failed to cause any blebbing in human cells (Figure S5C), suggesting that the requirement for this protein for cortical stability is not conserved between flies and humans, or that this function of SNX9 is redundant with its paralogs SNX18 and SNX33 in mammalian cells [48]. Together, our results suggest that IPIP27A acts together with pacsin 2 for successful cytokinesis, and that SH3PX1 and pacsin 2, which both associate with actin and contain BAR and SH3 domains, operate in a functionally analogous way to support IPIP function in *Drosophila* and mammals, respectively.

### dRab35 Is Also Required for PtdIns(4,5)P_2_ Homeostasis and Cortical Stability during Cytokinesis

Rab35 can recruit OCRL1 to newly forming endocytic vesicles, where it contributes to removal of PtdIns(4,5)P_2_ and membrane actin post-scission [51]. It was therefore of interest to examine the functional relationship between dRab35 and dIPIP in controlling dOCRL-dependent PtdIns(4,5)P_2_ homeostasis and cytokinesis. Depletion of dRab35 reduced endosomal targeting of dOCRL, in agreement with previous work (Figure S6A) [51]. Next, we could show that dRab35 depletion resulted in very similar phenotypes to those seen upon depletion of dIPIP or dOCRL. The Rab35-depleted cells displayed increased multinucleation, which has been reported previously [52], and which could be rescued by dRab35 re-expression (Figure S6B), accumulation of PtdIns(4,5)P_2-_containing vacuoles (Figure S6C) that also contained actin, Rho1 and dMoesin (Figures S6A, S6B, and S6D), and cortical membrane blebbing (Figure S6E). Co-depletion of dRab35 and dIPIP gave a similar degree of membrane blebbing to that seen with dIPIP alone (Figure S6F), and the degree of multinucleation was the same as that seen with depletion of either protein alone (Figures S6G, 1A, and S6B). Hence, both membrane recruitment, mediated by dRab35, and scaffolding to SH3PX1, mediated by dIPIP, are required for dOCRL function in PtdIns(4,5)P_2_ homeostasis, and normal cell division.

## Discussion

Here, we identify a role for IPIP27 in PtdIns(4,5)P_2_ homeostasis that is conserved from flies to man. IPIP27 functions as a scaffolding protein to link OCRL to BAR domain proteins, which is crucial for maintaining cellular PtdIns(4,5)P_2_ homeostasis, and in turn is important for cortical actin stability in cytokinesis. In *Drosophila*, the relevant IPIP27 partner is SH3PX1, whereas in human cells it is pacsin 2. Both proteins are present in endocytic intermediates [50, 53, 54], as are IPIP27 and OCRL [24, 29, 34, 54], consistent with an interaction between these proteins on endocytic structures, and a role in promoting the OCRL-dependent removal of PtdIns(4,5)P_2_ from newly forming endocytic vesicles [28, 29, 51]. Failure to remove PtdIns(4,5)P_2_ at this stage would result in its accumulation on endosomal compartments, as is observed upon dIPIP or dOCRL depletion. The same phenotype is also seen upon depletion of dRab35, which is required for OCRL recruitment to endocytic vesicles [51]. Hence, it is both membrane recruitment of OCRL, mediated by Rab35, and its further engagement with actin-associated BAR domain proteins, mediated by IPIP27, likely in regions of high membrane curvature, that is necessary for efficient PtdIns(4,5)P_2_ hydrolysis and the cellular homeostasis of this lipid.

Plasma membrane blebbing, as we observe in IPIP27-depleted cells, is reminiscent of the phenotypes seen upon depletion of actin nucleators [55] or actin-membrane linker proteins [13, 56, 57], consistent with the view that it is an actin phenotype [42]. Although blebbing is evident in both *Drosophila* and human cells, cytokinesis failure is only seen in *Drosophila* cells. However, both phenotypes can be rescued by correcting PtdIns(4,5)P_2_ homeostasis or actin stability, strongly suggesting a common underlying mechanism. We attribute the phenotypic differences between species to a lack of functional redundancy in *Drosophila* compared to humans. *Drosophila* has single orthologs of IPIP27 and OCRL, whereas humans have two paralogs of each (IPIP27A and IPIP27B, and OCRL and INPP5B), and humans express a larger number of inositol 5-phosphatases compared to *Drosophila*, including others within the endocytic pathway [47], which offers additional scope for functional redundancy or compensation in this species.

Our results strongly support the view that IPIP27 is acting indirectly during cytokinesis through the control of PtdIns(4,5)P_2_ homeostasis on endocytic membranes. However, it remains possible that it may also have a more direct role, possibly in the later stages of this process. In mammalian cells, Rab35 can promote OCRL1 recruitment to the midbody region during late cytokinesis for actin clearance and abscission [14]. It is therefore possible that IPIP27 may engage with OCRL, and possibly with BAR domain proteins, to promote OCRL activity at this stage. Because IPIP27 functions in the endocytic pathway, another possibility is that it participates in trafficking into the cleavage furrow or intercellular bridge, which is required for delivery of certain factors involved in cytokinesis to these regions [58]. However, the cytokinetic proteins we see accumulate on internal membranes are not typical endocytic cargoes, and the profound blebbing we see is not typically observed upon perturbation of endocytic traffic in cytokinesis [58]. Hence, our results are more consistent with an indirect involvement, with IPIP27 scaffolding function being critical for PtdIns(4,5)P_2_ homeostasis on endomembranes, which in turn is required for cortical membrane stability during cytokinesis.

## STAR★Methods

### Key Resources Table

**Table:** 

REAGENT or RESOURCE	SOURCE	IDENTIFIER
**Antibodies**		

Rabbit polyclonal anti-dIPIP	Laboratory of Sean Sweeney/This paper	N/A
Rabbit polyclonal anti-dOCRL	Laboratory of Sébastien Carréno	N/A
Rabbit polyclonal anti-dMoesin	[59]	N/A
Rabbit polyclonal anti-SH3PX1	[50]	RRID:AB_2567978
Rabbit polyclonal anti-Rab5	Abcam	Cat# ab13253; RRID:AB_299796
Rabbit polyclonal anti-Rab11	[60]	RRID:AB_2569806
Rabbit polyclonal anti-syndapin	[61]	RRID:AB_2569796
Sheep polyclonal anti-IPIP27A	[35]	N/A
Sheep polyclonal anti-IPIP27B	[35]	N/A
Sheep polyclonal anti-OCRL1	[22]	N/A
Sheep polyclonal anti-pacsin2	[35]	N/A
Sheep polyclonal anti-myosin1E	[35]	N/A
Rabbit polyclonal anti-SNX9	Sigma-Aldrich	Cat# HPA031410; RRID:AB_10603339
Rabbit polyclonal anti-GM130	[62]	N/A
Mouse monoclonal anti-Rho1 (clone p1D9)	DSHB	Cat# p1D9; RRID:AB_528263
Mouse monoclonal anti-Rab7	DSHB	Cat# Rab7; RRID:AB_2722471
Mouse monoclonal anti-Myc (clone 9B11)	Cell Signaling Technology	Cat# 2276; RRID:AB_331783
Mouse monoclonal anti-actin (clone C4)	BD Biosciences	Cat# 612656; RRID:AB_2289199
Mouse monoclonal anti-EEA1 (clone 14)	BD Biosciences	Cat# 610456; RRID:AB_397829
Mouse monoclonal anti-α-tubulin (clone DM1A)	Sigma-Aldrich	Cat# T9026; RRID:AB_477593
Mouse monoclonal anti-GAPDH (Clone G-9)	Santa Cruz Biotechnology	Cat# sc-365062; RRID:AB_10847862

**Bacterial and Virus Strains**		

*Escherichia coli* BL21-codon plus RIPL cells	Agilent technologies	Cat#NC9122855

**Chemicals, Peptides, and Recombinant Proteins**		

Alexa 488-conjugated phalloidin	Thermo Fisher Scientific	Cat# A12379; RRID:AB_2315147
Alexa 647-conjugated phalloidin	Thermo Fisher Scientific	Cat# A12381; RRID:AB_2315633
Rhodamine-conjugated phalloidin	Thermo Fisher Scientific	Cat# R415; RRID:AB_2572408
Phalloidin-TRITC	Sigma-Aldrich	Cat# P1951; RRID:AB_2315148
Goat anti-mouse IgG (H+L) Alexa 488	Thermo Fisher Scientific	Cat# A-11001; RRID:AB_2534069
Goat anti-rabbit IgG (H+L) Alexa 488	Thermo Fisher Scientific	Cat# A-11008; RRID:AB_143165
Goat anti-rabbit IgG (H+L) Alexa-647	Thermo Fisher Scientific	Cat# A-21244; RRID:AB_2535812
Donkey anti-rabbit IgG ECL HRP	GE Healthcare	Cat# NA9340-1ml; RRID:AB_772191
Sheep anti-mouse IgG ECL HRP	GE Healthcare	Cat# NA931; RRID:AB_772210
Donkey anti-sheep IgG HRP	Santa Cruz Biotechnology	Cat# sc-2473; RRID:AB_641190
Vectashield antifade mounting medium with DAPI	Vector Laboratories	Cat#H-1200
Schneider’s Drosophila medium	Thermo Fisher Scientific	Cat# 21720024
Dulbecco’s modified Eagle’s medium	Sigma-Aldrich	Cat#D6429
Heat-inactivated fetal bovine serum	Thermo Fisher Scientific	Cat#16140071
Penicillin-Streptomycin	Sigma-Aldrich	Cat#P4333
L-glutamine	Sigma-Aldrich	Cat#G7513
FugeneHD transfection reagent	Promega corporation	Cat#E2311
INTERFERin	Polyplus transfection	Cat#409-10
Opti-MEM	Thermo Fisher Scientific	Cat#31985062
Latrunculin A	Sigma-Aldrich	Cat#428021
Jasplakinolide	Sigma-Aldrich	Cat#J4580
Hygromycin B	Thermo Fisher Scientific	Cat#10687010
*m*-3M3FBS	Tocris Bioscience	Cat#1941
*o*-3M3FBS	Tocris Bioscience	Cat#1942
Pierce ECL Western Blotting Substrate	Thermo Fisher Scientific	Cat#34577

**Critical Commercial Assays**		

pENTR/D-TOPO Cloning Kit	Thermo Fisher Scientific	Cat#K240020
T7 RiboMAX Express Large Scale RNA Production System	Promega corporation	Cat#P1320
Gateway LR clonase enzyme mix	Thermo Fisher Scientific	Cat#11791019

**Experimental Models: Cell Lines**		

Schneider’s S2 cells	Laboratory of Sébastien Carréno	N/A
HeLa cell line	ATCC	Cat# CRM-CCL-2; RRID:CVCL_0030
S2 expressing GFP-Utrophin-CH/GFP-Tubby and mCherry-tubulin	[31]	N/A
S2 expressing anilllin-mCherry and GFP-Spaghetti-squash	Laboratory of Gilles Hickson	N/A
HeLa expressing Lifeact-GFP	This paper	N/A

**Oligonucleotides**		

siRNA targeting sequence: IPIP27A_siRNA1: GGUGACAGACUCAGCCCAA	Dharmacon	Cat#J-015976-12-0005
siRNA targeting sequence: IPIP27A_siRNA2: AGGGCGAUCUGUGGCCUGAAA	QIAGEN	Cat#Hs_FAM109A_2
SMARTpool of 4 siRNAs targeting IPIP27B: GGAGCUUGGACACGGGAUU, CUGGCUGGGCUCCGGUAAA, GGGAAUGUCACCCGAGACU, UGGCCGAAGAUGCUGGUUU	Dharmacon	Cat#L-024579-01-0005
SMARTpool of 4 siRNAs targeting CD2AP: GAAUUGUUGUGCAUUGUAG, AACUAAAGCUAGAUUCUGA, GUAAGGACCUCCAAAGAAA, UGACAUAGCUUCCUCAGAA	Dharmacon	Cat#L-012799-00-0005
siRNA targeting sequence: OCRL1_siRNA1: GAACGAAGGUACCGGAAAG	Dharmacon	Cat#J-010026-07-0005
siRNA targeting sequence: OCRL1_siRNA2: CGAAGAAGACUAAGGCUUU	Dharmacon	Cat# J-010026-08-0005
siRNA targeting sequence: pacsin2_siRNA1: CCCUUAAUGUCCCGAGCAA	Dharmacon	Cat#J-019666-19-0005
siRNA targeting sequence: pacsin2_siRNA2: CCUCACUGAUGAACGAUGA	Dharmacon	Cat#J-019666-20-0005
siRNA targeting sequence: INPP5B: GGACAAGGCUCAUAUUUUA	Dharmacon	Cat#J-021811-09-0005
siRNA targeting sequence: myosin1E: GUUCAAGGGUGUAAAGCGA	Dharmacon	Cat#J-019919-10-0005
siRNA targeting sequence: SNX9: UAAGCACUUUGACUGGUUAUU	Dharmacon	Cat#J-017335-05-0005

**Recombinant DNA**		

cDNA clone SD10969 (CG12393-dIPIP)	Drosophila Genomics Resource Center	DGRC:4231 Flybase Id: FBcl0275167 http://flybase.net/reports/FBcl0167040.html
cDNA clone LD21953 (CG9575- Rab35)	Laboratory of Vincent Archambault	DGRC:7308 Flybase Id: FBcl0167040
pAc5.1/V5-His A	Thermo Fisher Scientific	Cat t#V411020
pAc5.1/mRuby	Laboratory of Richard Baines	N/A
pAc5.1-dIPIP-mRuby	This paper	N/A
pAc5.1-Myc-dIPIP	This paper	N/A
pAc5.1-Myc-dIPIP F267A	This paper	N/A
pAc5.1-Myc-dIPIP ^156^RR^157^ > AA	This paper	N/A
pAc5.1-Myc-dIPIP ^293^LIQL^296^ > AAAA	This paper	N/A
pAc5.1-Myc-IPIP27A	This paper	N/A
GST-dIPIP	This paper	N/A
pAC5.1-GFP-dOCRL	[31]	N/A
pAC5.1-GFP-dOCRL G365E	[31]	N/A
RFP-SH3PX1	Laboratory of Graydon Gonsalvez [50];	N/A
pMT-Ch-W	Drosophila gateway vector collection	N/A
pMT-mCherry-dRab35	This paper	N/A
pEGFP-C1 Lifeact-EGFP	Laboratory of George Banting	RRID:Addgene_58470

**Software and Algorithms**		

Image Lab Software	Bio-Rad http://www.bio-rad.com/en-ca/sku/1709690-image-lab-software?ID=1709690	RRID: SCR_014210
FIJI (ImageJ version 2.0.0-rc-24/1.49 m)	NIH http://fiji.sc	RRID:SCR_002285
Volocity version 6.3	Perkin Elmer http://www.perkinelmer.com/pages/020/cellularimaging/products/volocity.xhtml	RRID:SCR_002668
GraphPad Prism 7	GraphPad http://www.graphpad.com/	RRID:SCR_002798
MetaMorph Microscopy Automation and Image Analysis Software	Molecular devices http://www.moleculardevices.com/Products/Software/Meta-Imaging-Series/MetaMorph.html	RRID:SCR_002368
NIS-Elements	Nikon Instruments https://www.nikoninstruments.com/Products/Software	RRID:SCR_014329
Huygens Software	Scientific Volume imaging https://svi.nl/HuygensSoftware	RRID:SCR_014237
Adobe photoshop CS6	Adobe https://www.adobe.com/products/photoshop.html	RRID:SCR_014199
Adobe illustrator CS6	Adobe http://www.adobe.com/products/illustrator.html	RRID:SCR_010279

### Contact for Reagent and Resource Sharing

Further information and requests for resources and reagents should be directed to and will be fulfilled by the Lead Contact, Martin Lowe (martin.lowe@manchester.ac.uk).

### Experimental Model and Subject Details

#### Cell culture

*Drosophila* Schneider 2 (S2) cells (derived from a primary culture of late-stage 20-24 hour old *Drosophila melanogaster* embryos) were cultured at 27°C in Schneider’s *Drosophila* medium (Life Technologies) supplemented with 10% (v/v) heat-inactivated FBS (Thermo Fisher Scientific), 50000 units of Penicillin and 50 mg/ml Streptomycin (Sigma). S2 cells stably expressing mCherry-tubulin with either GFP-Tubby or GFP-Utrophin-CH [31] anillin-mCherry with Spaghetti-squash-GFP or anillin-GFP only were cultured at 27°C in medium containing 0.3 mg/ml Hygromycin-B (EMD Millipore). Expression of anillin-mCherry with Spaghetti-squash-GFP or anillin-GFP, all under control of the metallothionein promoter, was induced with 0.5 mM of copper sulfate 24 hours prior to imaging. HeLa cells (derived from cervical cancer cells taken from a human female suffering from cervical cancer) were cultured in DMEM supplemented with 10% (v/v) heat-inactivated fetal bovine serum (Thermo Fisher Scientific) and 1 mM L-glutamine at 37°C and in 5% CO_2_. Cultured HeLa cells were routinely tested for mycoplasma contamination by DNA staining with Hoechst 33342 and by PCR (EZ PCR Mycoplasma test kit, Geneflow).

### Method Details

#### Molecular Biology

The CG12393 cDNA sequence encoding dIPIP (DGRC clone SD10969) was amplified by PCR to include an N-terminal Myc tag and subcloned into the pAc5.1-V5-His vector (Invitrogen). dIPIP C-terminally tagged with mRuby was generated by cloning dIPIP cDNA into a modified pAc5.1-V5-His A vector (obtained from Richard Baines’ lab, Manchester). dIPIP cDNA was cloned into the pGEX-6P-1 vector to generate a GST fusion. Human Myc- tagged IPIP27A cDNA was cloned into the pAc5.1-V5-His vector. GFP- and mCh-dRab35 constructs were generated by PCR amplification of the ORF from cDNA (DGRC Clone LD21953, Vincent Archambault’s lab, Montreal), followed by cloning into pENTR-D-TOPO (Invitrogen) followed by recombination using LR Clonase into the pMT-ChW destination vector (*Drosophila* Gateway Vector Collection; T. Murphy, Carnegie Institution for Science, Washington, DC). Mutagenesis was performed using the Quikchange site-directed mutagenesis method (Agilent technologies). All constructs were verified by DNA sequencing (GATC Biotech).

#### DNA transfections

Transient and stable DNA transfections were performed with FugeneHD (Promega Corporation) according to manufacturer’s instructions using a 3:1 FugeneHD to DNA ratio. Transient transfections in S2 cells (seeded at a density of 3.0x10^5^ cells/250 μl onto sterile 12 mm glass coverslips and incubated overnight at 27°C) were performed with FugeneHD. 0.4 μg or 1.0 μg of DNA was transfected for rescue experiments and localization experiments respectively and cells were incubated for 48 hours at 27°C prior to fixation and analysis. For inducible transiently expressed constructs, induction with 0.5 mM Copper Sulfate was performed 10 hours after transfection. A stable HeLa cell line constitutively expressing Lifeact-GFP was generated by transfecting 3.0x10^6^ cells with 12 μg of the pEGFP-C1 LifeAct-EGFP plasmid (George Banting, University of Bristol, UK). Resistant cells were selected in 1 mg/ml G418 for 48 hours after transfection, and high-expressing cells were sorted by FACS (FACSAria Fusion, BD).

#### RNA interference

RNA interference was performed in S2 cells using double stranded RNAs (dsRNA). Between 200-600 bp DNA template was amplified from cDNA or genomic DNA (in the case of UTR regions) by PCR using gene-specific primers that included the T7 promoter sequence, and subsequently used for *in vitro* transcription to generate dsRNA (T7 RiboMAX Express, Promega Corporation). dIPIP RNAi_2 (targeting the 3′UTR) was used for all depletion-rescue experiments. dIPIP RNAi_1 was generated using primer sequences; 5′-TAATACGACTCACTATAGGGAGGTCAACAAGGCCTTCCA-3′ and 5′-TAATACGACTCACTATAGGGACGTCTCCATGCTGTCTTGG-3′, dIPIP RNAi_2 was generated using primer pair 5′- TAATACGACTCACTATAGGGATTCCGCACCCAGCAATCGATAACC-3′ and 5′-TAATACGACTCACTATAGGGGGCTGCACTGTCTCGGGCTGC-3′. dOCRL_RNAi_1 was generated using primer pair: 5′- TAATACGACTCACTATAGGGAGAAAGGACATTGTCAAGGAGCGC-3′ and 5′-TAATACGACTCACTATAGGGAGAATCGCGTAGATATCCGGCGGC-3′ and dOCRL_RNAi_2 was generated using primer sequences: 5′- TAATACGACTCACTATAGGGGATTGCCAATAATTGTCATCGC-3′ and 5′- TAATACGACTCACTATAGGGGATTCTGAG TACTGATAGGG-3′. dsRNAs against SH3PX1 and dRab35 were generated using primer pairs; 5′- TTAATACGACTCACTATAGGGAGAGGCCATCTCGCCGCCGG-3′ and 5′- TTAATACGACTCACTATAGGGAGACTGACGGCTGGCCTCCT-3′ [63] and 5′- TAATACGACTCACTATAGGGATGAAATATTTTCGGCACCAAATCGCCGTC-3′ and 5′-TAATACGACTCACTATAGGGAGCTGCTGTTGCTGATGTTTTTGTTGCTGT-3′ respectively. For fixed sample imaging of depleted cells, 1.2x10^5^ S2 cells were cultured on glass coverslips in 50% (v/v) of serum free medium and treated with the relevant dsRNA for 6 days. dsRNA (1.5 μg/well or 3.5 μg/well of a 96- or 24-well plate respectively) was added directly to the cells in fresh serum-free medium on day 0 and day 3. 50% (v/v) of complete medium was added to the cells after 20 minutes of incubation with dsRNA in serum-free medium. For live-cell time lapse imaging, 1.2x10^5^ S2 cells were treated with dsRNA for 6 days and transferred into a 4 or 8-well chamber slide (Ibidi) an hour prior to imaging. RNA interference in HeLa cells was performed using Interferin (Polyplus Transfection) and suitable siRNA oligos according to manufacturer’s instructions. pGL2 Firefly Luciferase (CGUACGCGGAAUACUUCGA) (Eurogentec) was used as a negative control. IPIP27A was targeted with 15 nM of an oligo derived from a Dharmacon SMARTpool (GE Healthcare Dharmacon) (referred to as siRNA_1), or 12 nM of the Hs_FAM109A_2 oligo from the Flexitube Genesolution package (QIAGEN) (referred to as siRNA_2). OCRL1, INPP5B, pacsin 2 and myosin1E, were also targeted with single oligos derived from a Dharmacon SMARTpool while IPIP27B and CD2AP were targeted with complete Dharmacon SMARTpools. All siRNA target sequences are listed in the Key Resources Table. HeLa cells were plated in 35 mm dishes at a density of 3.5x10^4^ cells/ml 24 h prior to siRNA transfection, and RNA interference was carried out for a further 72 h.

#### Drug treatments

For drug treatments, S2 cells were depleted of the protein of interest for 6 days and transferred at a density of 1.0x10^6^ cells/ml onto 4-well chamber slides followed by treatment with the relevant drug. HeLa cells were depleted of the protein of interest for 48 hours and then split at a density of 2.4x10^4^ cells/ml and incubated overnight before drug treatments. Latrunculin A (LatA), Jasplakinolide (JAS) (Sigma-Aldrich), a PLC agonist (*m*-3M3FBS) and a PLC analog (*o*-3M3FBS) (Tocris Bioscience) were diluted in complete growth medium and added to cells for 2 hours prior to the start of live imaging and cells were imaged in the presence of the drugs throughout the duration of the time-lapse. An equal volume of the respective drug solvent (DMSO for LatA and JAS) or Ethanol (PLC agonist and PLC analog) was added alongside to cells of each condition as a control. When PLC agonist and PLC analog were used in rescue experiments in S2 cells, the drugs were added at a final concentration of 50 μM straight to cells 4 days after protein depletion and incubated for a further 2 days. The cells were split and placed in fresh media prior to the start of imaging.

#### Immunofluorescence microscopy

S2 cells were grown on 12mm coverslips and fixed in 4% paraformaldehyde for 20 minutes at room temperature. Cells were washed in TBS (20 mM Tris HCl pH 7.6, 150 mM NaCl) and blocked for 30 minutes in blocking buffer (5% normal goat serum in TBS containing 0.1% Triton X-100 (TBST) for 1 hour at room temperature. Incubation with primary antibodies was performed for 2 h at room temperature (for SH3PX1) or overnight at 4°C (for all remaining antibodies) in TBST. Following incubation with Alexa Fluor-conjugated secondary antibodies (Thermo Fisher Scientific) for 2 hours at room temperature, coverslips were mounted in Vectashield mounting medium with DAPI (Vector Laboratories) and visualized on a DeltaVision fluorescence microscope (Applied Precision) using a 60x/ 1.42 Plan Apo oil objective. Images were collected with a Z optical spacing of 0.2 μm using a CoolSNAP HQ (Photometrics) camera with MetaMorph software (Molecular Devices). Predictive deconvolution was then carried out on raw images using Huygens software (Scientific Volume Imaging). Quantification of multi-nucleation was carried out in FIJI (National Institutes of Health). All images were adjusted for contrast using Adobe Photoshop CS6 and figures were assembled using Adobe illustrator CS6.

#### Live cell imaging

S2 cells were depleted of the protein of interest for 6 days then split into a 4 or 8-well chamber slide (Ibidi) an hour prior to imaging. Cells were maintained at 25°C in an environmentally controlled chamber and imaged over 10-16 h. Brightfield and fluorescence images were acquired using a CoolSNAP HQ2 (Photometrics) camera on a Nikon Ti-E inverted microscope using a 60x/1.4 Plan Apo oil objective equipped with a Perfect Focus System (PFS) and driven by NIS Elements software (Nikon Instruments Inc.). Time-lapse imaging of S2 cells expressing MRLC-GFP and anillin-mCherry cells was performed using a DeltaVision microscope (Applied Precision) equipped with a CoolSnap HQ2 camera (Photometrics) and with a 60x/1.42 planApo objective with a Z optical spacing of 2 μm. Deconvolution was carried out using the softWoRx software (Applied Precision). Time-lapse imaging of S2 cells expressing GFP-Tubby was performed using a spinning-disc confocal system (Ultra-VIEW Vox; PerkinElmer) using a scanning unit (CSU-X1; Yokogawa Corporation of America) and a CCD camera (ORCA-R2; Hamamatsu Photonics) fitted to an inverted microscope (DMI6000 B; Leica) equipped with a motorized piezoelectric stage (Applied Scientific Instrumentation). Image acquisition was performed using Volocity version 6.3 (PerkinElmer) using Plan Apochromat 63x oil immersion objectives, NA 1.4, with a Z optical spacing of 0.5 μm and with camera binning set to 2x2. Montages of time-lapse videos were generated in FIJI (National Institutes of Health). HeLa cells were depleted of the protein of interest for 2 days after which they were split into fresh medium at a density of 2.5x10^4^ cells/ml in a 6- or 12-well plastic culture dish (Corning Inc.) and subjected to time-lapse live imaging 3 days after protein knock down. Cells were maintained at 37°C and 5% CO_2_ in an environmental control chamber (Solent Scientific). Phase contrast or fluorescence images were acquired every 5-6 minutes over 16-18 h using constant exposure parameters, on an AS MDW live cell imaging system (Leica Microsystems, Wetzlar Germany) using a 20x/ 1.30 Plan Apo glycerine objective. The microscope was equipped with an automated stage (PZ-2000; Applied Biosystems) and point visiting was used to allow multiple positions to be imaged within the same time course. The images were collected using a CoolSNAP HQ camera (Photometrics) and Imaging software Image Pro 6.3 (Micromanager Media Cybernetics Ltd).

#### Antibody generation and affinity purification

Polyclonal antibodies to dIPIP were generated in rabbits using GST-tagged full-length dIPIP as immunogen, and antibodies were affinity purified against this protein. A polyclonal antibody to dOCRL was raised against the amino terminus of dOCRL (amino acids 1-183) and generated in rabbits.

#### Recombinant protein expression

Recombinant GST-tagged proteins were expressed and purified from BL21 *Escherichia coli* cells. Cells were grown in LB medium at 37°C to an optical density of 0.6 at A_600_ and recombinant protein expression induced by addition of 0.1 mM isopropyl b-D-1-thiogalactopyranoside (IPTG) and further incubation at 18°C overnight. Cells were resuspended in lysis buffer (20 mM HEPES pH 7.4, 0.2 M NaCl,1 mM EDTA, 5 mM MgCl_2_, 0.1% Triton X-100, 1 mg/ml lysozyme, 10 μg/ml DNase) containing and protease inhibitors (Cocktail inhibitor set III, Calbiochem) and lysed by a freeze-thaw cycle, consisting of snap freezing in liquid nitrogen followed by incubation at 37°C for 10 min. Bacterial lysates were cleared by centrifugation at 13 000 rpm for 20 min at 4°C and applied to glutathione-Sepharose beads (GE Healthcare) for 3 h at 4°C with rotation. Following washing with column buffer (20 mM HEPES pH 7.4, 0.2 M NaCl, 1 mM DTT), GST-tagged proteins were eluted by addition of column buffer containing 50 mM glutathione and desalted on PD-10 columns (GE Healthcare). Following addition of 10% (v/v) glycerol, proteins were snap frozen in liquid nitrogen and stored at −80°C.

#### Cell extract preparation

S2 cells (growing in 6-well dishes) were harvested by centrifugation after 6 days protein knock down and the cells were washed twice in ice cold PBS prior to lysis in 100 μl/dish HMNT (20 mM HEPES pH 7.4, 5 mM MgCl_2_, 0.1 M NaCl, 0.5% Triton X-100 plus Protease Inhibitor Cocktail III (Sigma-Aldrich)) by incubation on ice for 30 min with vortexing every 5 min. Extracts were clarified by centrifugation at 13K rpm for 15 min at 4°C in a microfuge. Extracts were prepared from HeLa cells (growing in 3.5 cm dishes) by washing the cells twice in cold PBS for 5 min and extraction in 150 μl/dish of HMNT for 15 min on ice with shaking. Extracts were clarified by centrifugation at 13K rpm for 15 min at 4°C in a microfuge.

#### Protein binding experiments

S2 cell extract (500 μl/pull down at 2.5 mg/ml) was cleared by ultra-centrifugation at 55,000 rpm for 20 min at 4°C and incubated with glutathione-Sepharose beads containing GST-tagged bait protein (40 μg/pull down) for 4 h at 4°C with shaking. After binding, beads were centrifuged at 4,500 rpm for 5 min at 4°C and the unbound fraction collected. Beads were washed 3 times with cold HMNT and the bound fraction was eluted by incubating the beads in 2X SDS sample buffer at 95°C for 10 min.

#### Immunoblotting

Samples were subjected to SDS-PAGE, transferred to Amersham Protran nitrocellulose membrane using constant current (300 mA) for 2.5 hours. Membranes were blocked in 5% milk in PBS containing 0.15% Tween-20 (PBST) for 45 min at room temperature and incubated in blocking solution containing primary antibody overnight at 4°C. Membranes were washed in PBST and incubated with the relevant HRP-coupled secondary antibody in blocking solution for 1 h at room temperature, followed by washing in PBST and signal development with ECL SuperSignal West Pico chemiluminescence substrate (Thermo Fisher Scientific) and visualization on a ChemiDoc MP imaging system (Bio-Rad, UK).

#### Experimental design

##### Sample size and replication

For quantification of multinucleation and cells harboring PtdIns(4,5)P_2-_rich vacuoles, at least 500 cells were quantified per condition per experimental repeat. At least 3 independent biological replicates were carried out for each experiment and every condition was set up in duplicate per experiment. For live cell imaging in S2 and HeLa cells at least 50 or 100 cells respectively, were quantified per condition per experimental repeat. At least 3 experimental repeats were performed for every experiment. The number of cells analyzed in each experiment is indicated in the respective figure legend.

##### Randomization

For quantification of multinucleation and cells harboring PtdIns(4,)P_2_-rich vacuoles, fields of view were selected randomly during quantification while ensuring (i) non-overlapping fields of view and (ii) that cells in at least 5 fields of view spanning the entire coverslip were quantified that is, 2 fields in the extreme top and bottom right of the coverslips, a field in the center of the coverslip and another 2 fields in the extreme top and bottom left of the coverslip. The order of visualization of these fields was random in each condition.

##### Inclusion and exclusion criteria of any data

In the case of rescue experiments, the coverslip was carefully scanned laterally starting from top left through to bottom right to visualize all transfected cells. Only the transfected cells that displayed fluorescence intensity at least twice above background intensity in the respective channel were included in the count. For live cell imaging data using HeLa cells, cell divisions were analyzed and scored for phenotypes 3 days after RNAi knock down of the respective protein. Only cells that were in metaphase (as indicated by the cell’s rounded shape) at the start of imaging were included in the count. For live imaging in S2 cells, all cell divisions were analyzed and scored for phenotypes after 6 days of protein knock down. Only dividing cells that started off as mononucleated were included in the count, and cells that were already binucleated and subsequently attempted to undergo a second round of division, were not counted.

### Quantification and Statistical Analysis

ImageJ was used to obtain fluorescence intensity values to determine the rescue ability of the various dIPIP and dOCRL constructs and to calculate the ratio of Rho1 and dMoesin at the plasma membrane versus endomembranes. Multinucleated cells were assessed by the presence of at least 2 nuclei in one cell, as labeled by Hoechst 33342 and the plasma membrane labeled with actin. To measure the time for actin dissolution in the contractile ring, movies were analyzed using FIJI and kymographs were generated from line scans drawn through the contractile ring and spanning the entire length of the dividing cell from one pole of a dividing daughter cell to the opposite pole of the other daughter cell. The time for actin dissolution was then measured from fluorescent intensity plots derived from the kymograph. The time point after which the fluorescence intensity values of three consecutive time points approached basal interphase values, was taken as the time point for the completion of actin dissolution. Colocalization analysis was performed in Volocity version 6.3 (Perkin Elmer). The thresholds were set manually by drawing a region of interest outside the cell that was analyzed.

All western blots were quantified using Image Lab software versions 5-6 (BioRad). All graphs were generated and statistical analyses conducted using GraphPad Prism 6-7 software (GraphPad Software). At least 3 independent replicates were carried out for each experiment (unless otherwise indicated in the figure legend). Statistical details (test used, p values and significance level) and the sample size (number of cells assessed per experimental repeat) of all experiments are indicated in the respective figure legends and significance is indicated using asterisks above bars in graphs displaying quantification in the figures. Gaussian distribution of data was assessed using the D’Agostino-Pearson test. Means were compared using an unpaired Student’s t test to analyze data with a normal distribution. A Chi-square test was conducted on categorical data (numbers of cells displaying the different phenotypes in knock down versus control siRNA or after drug treatments). In these cases, raw data was used for statistical analysis while graphs presented show percentages. Statistical differences in the time for actin dissolution between control and IPIP27A-depleted cells were assessed using the non-parametric Mann-Whitney test. A one-sample t test was used to compare the mean ratio of fluorescence intensity of Rho1 and dMoesin on endomembranes versus the plasma membrane in dOCRL- or dIPIP-depleted cells with the mean ratio in control cells. Statistical significance cut-offs were defined as: ^∗^p < 0.05, ^∗∗^p < 0.001, ^∗∗∗^p < 0.0001.

## Supplemental Information

Document S1. Figures S1–S6

Document S2. Article plus Supplemental Information
