# Supplementary material for: IPIP27 Coordinates PtdIns(4,5)P2 Homeostasis for Successful Cytokinesis
Source: Curr Biol. 2019 Mar 4;29(5):775–789.e7. doi: 10.1016/j.cub.2019.01.043 (PMC6408333; doi:10.1016/j.cub.2019.01.043)
Supplement: Document S1. Figures S1–S6 [file mmc1.pdf]

Current Biology, Volume *29*

Supplemental Information

IPIP27 Coordinates PtdIns(4,5)P_2 Homeostasis for Successful Cytokinesis

Sabrya C. Carim, Khaled Ben El Kadhi, Guanhua Yan, Sean T. Sweeney, Gilles R. Hickson, Sébastien Carréno, and Martin Lowe

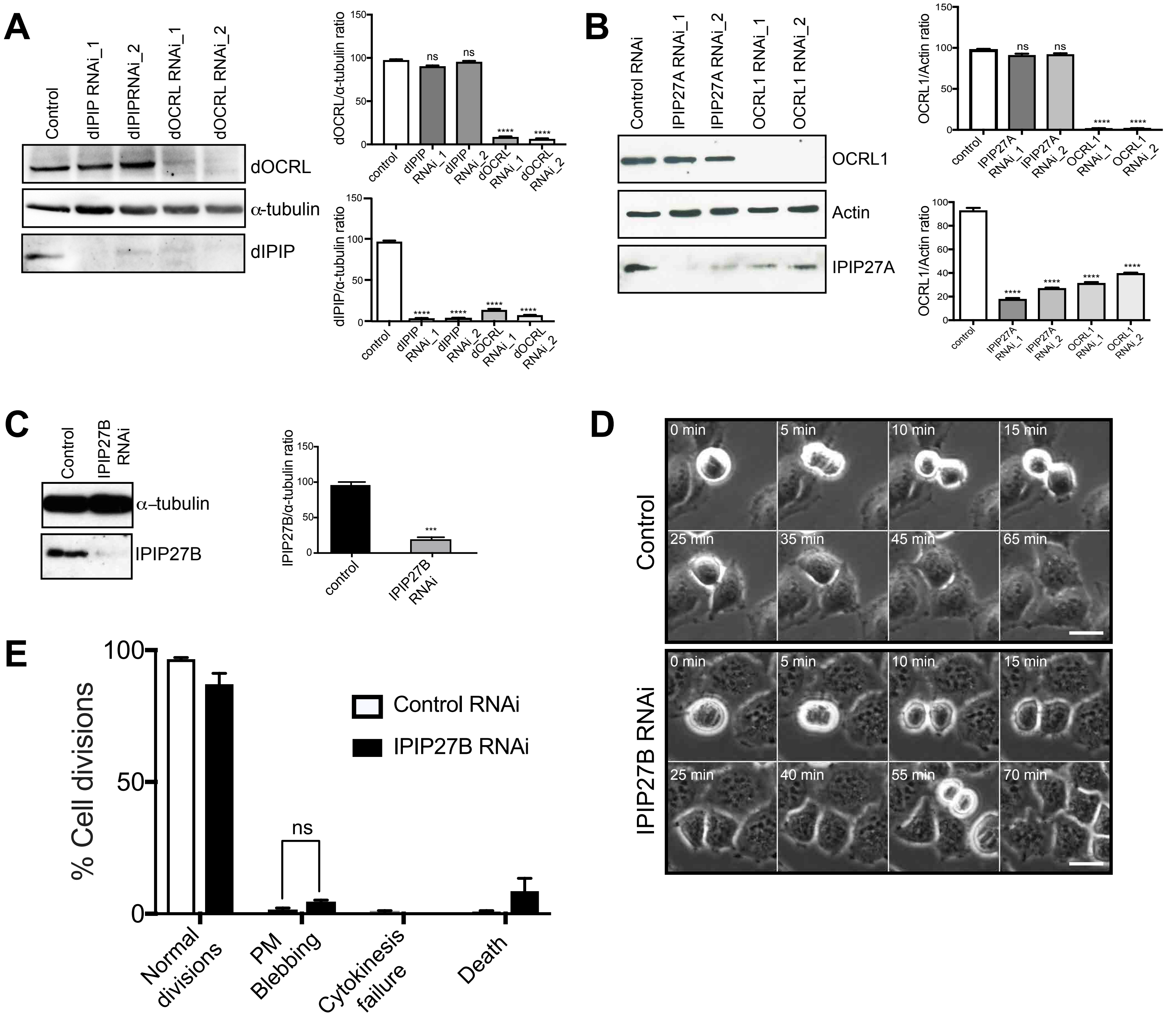

Figure S1. IPIP stabilization by OCRL and depletion of human IPIP27B. Related to Figures 1 and 4. (A) Western blot of protein extracts from S2 cells subjected RNAi for dIPIP or dOCRL probed with the indicated antibodies. (B) Western blot of protein extracts from control or RNAi-treated HeLa cells probed with the indicated antibodies. (C) Western blot showing the efficiency of IPIP27B depletion using a SMARTpool containing 4 siRNA duplexes targeting distinct sites within the gene. α-tubulin is a loading control Graphs in A,B,and C show quantification of the levels of dIPIP, dOCRL, IPIP27A, OCRL1 and IPIP27B before and after depletion.Values are means ± SEM of 3 independent experiments each done in triplicate, $^{**}p<0.005$, $^{***}p<0.0001$, Student's t-test. (D) Time-lapse live stills of control-depleted and IPIP27B-depleted cells during cell division. Scale bars, 10 µm. (E) Quantification of the percentage of cell divisions displaying the indicated phenotypes in control versus IPIP27B-depleted cells. Bars represent the mean and between 100-150 cells were analyzed per condition in 3 independent experiments. Error bars represent SEM. ns= non-significant, Chi-squared analysis.

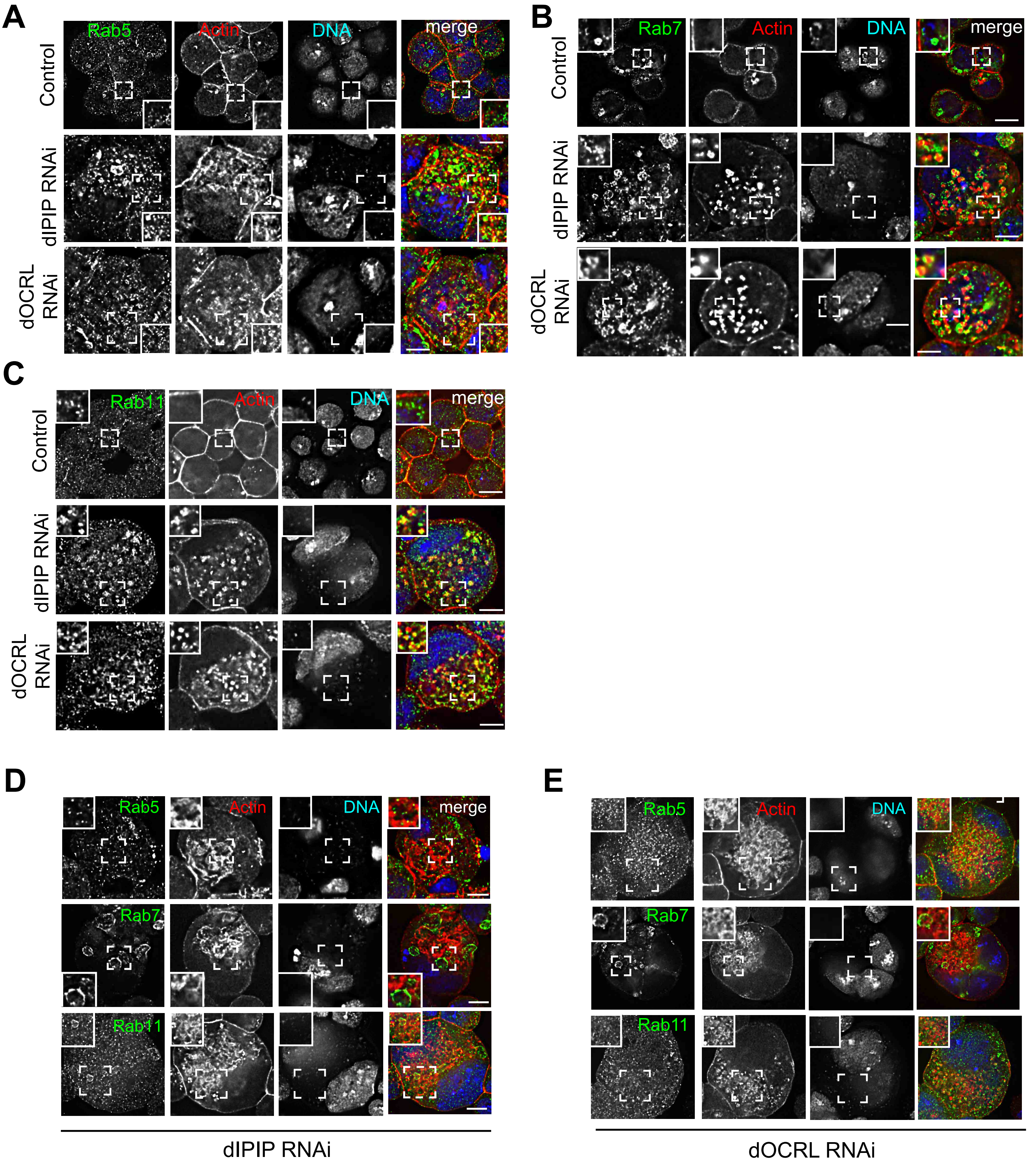

Figure S2. Altered endosome compartment morphology and actin accumulation upon dIPIP or dOCRL depletion. Related to Figure 2. (A-C) Control or dIPIP-depleted or dOCRL-depleted cells were analysed by immunofluorescence microscopyusing antibodies to endogenous Rab5, Rab7, or Rab11 (green), phalloidin to stain F-actin (red) and Hoechst 33342 to label DNA (blue). White boxes show zoomed insets and dashed white boxes represent the regions shown in zoomed insets. Scale bars, 5 µm.(D) and (E) S2 cells depleted of dIPIP or dOCRL were labeled with antibodies against Rab5, Rab7 or Rab11 (green) and phalloidin to detect F-actin (red). DNA was detected with Hoechst 33342 (blue). White boxes show zoomed insets and dashed white boxes represent the regios shown in zoomed insets. Sale bars, 5 µm. (D) and (E) show cells with larger F-actin rich vacuoles.

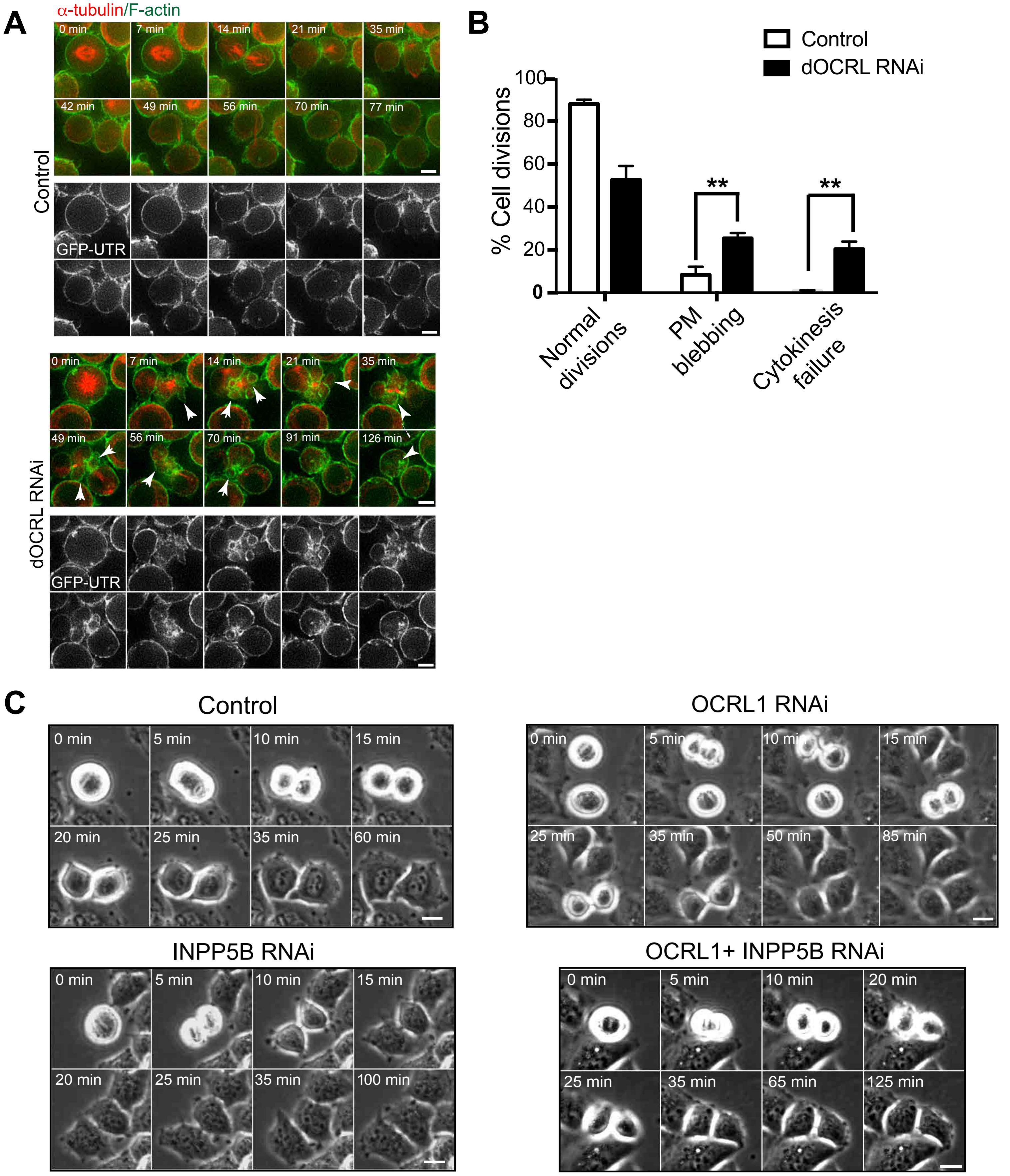

Figure S3. Depletion of dOCRL in S2 cells causes cortical blebbing and cytokinesis failure whereas depletion of OCRL1 and INPP5B in HeLa cells does not. Related to Figure 5. (A) Time-lapse stills showing dividing control or dOCRL-depleted S2 cells stably co-expressing GFP-Utrophin and mCherry-tubulin. White arrowheads indicate plasma membrane blebs. Scale bars, 5 μm. (B) Quantification of cell divisions displaying the indicated phenotypes. Bars represent the mean of 3 independent experiments with between 50-100 cells analyzed per condition per experiment. Error bars represent SEM. $^{**}p<0.001$ Chi-squared analysis. Time-lapse stills of dividing control, OCRL1-depleted, INPP5B-depleted HeLa cells, or Hela cells depleted for both OCRL1 and INPP5B. Scale bars, 10μm.

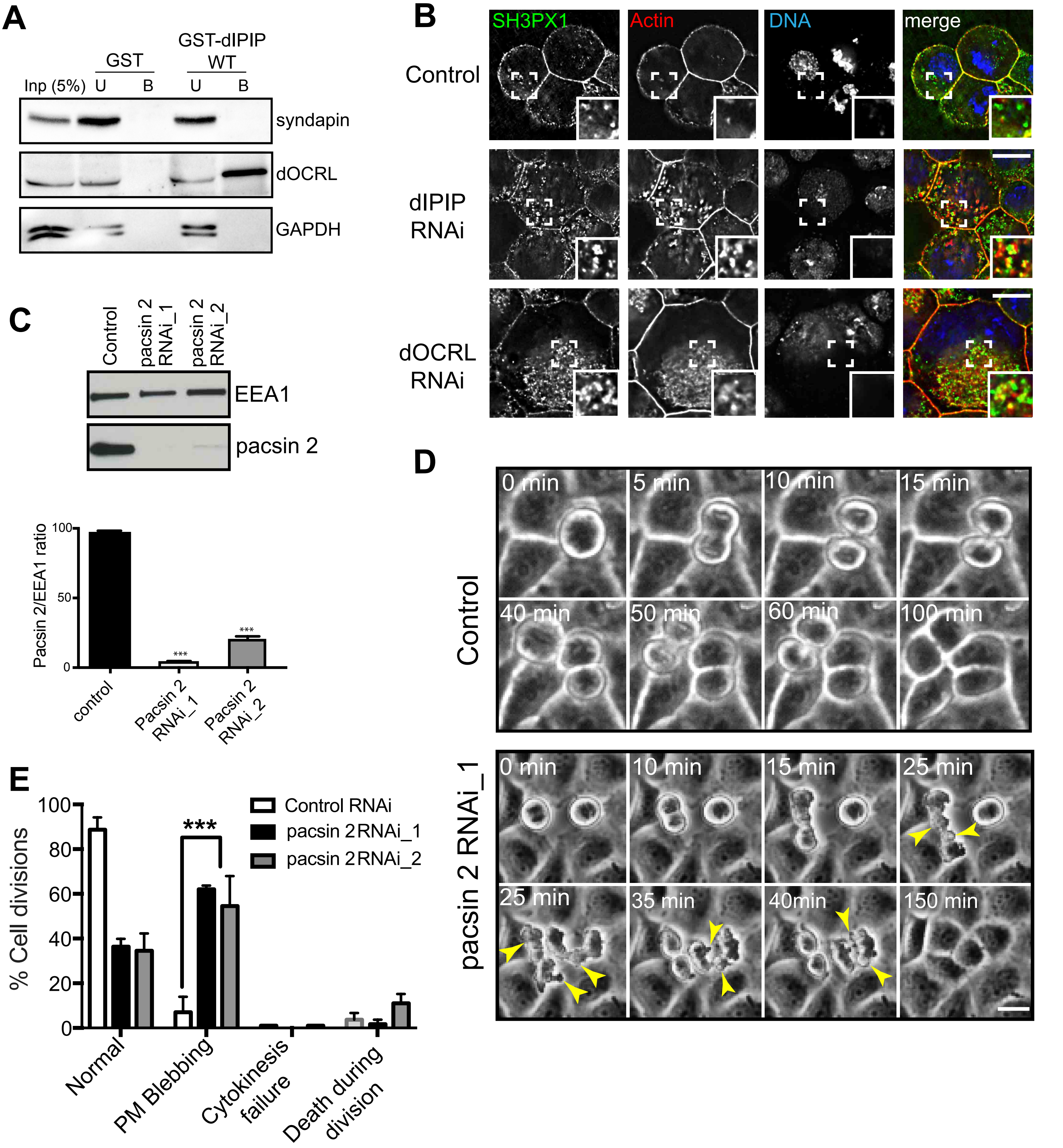

Figure S4. Analysis of dIPIP association with syndapin and SH3PX1, and depletion of human pacsin 2. Related to Figures 6 and 7. (A) Pull down assay using recombinant GST or GST-tagged wild-type (WT) full-length dIPIP as bait and S2 cell lysate. Samples were subjected to SDS-PAGE and Western blotting with the indicated antibodies. Inp, input (5%), U, unbound fraction (5%), B, bound fraction (50%). (B) S2 cells were depleted of dOCRL or dIPIP and fixed and stained with anti-SH3PX1 antibody, TRITC-phalloidin (F-actin) and Hoechst 33342 (DNA). White boxes show zoomed insets and dashed white boxes represent the region shown in zoomed insets. Scale bar, 5 µm. (C) Western blot showing pacsin 2 depletion from HeLa cells with 2 independent siRNAs. EEA1 is a loading control. Quantitation of the relative protein abundance following depletion. Values are means ± SEM of 3 independent experiments each done in triplicate,***$p<0.0001$, Student's t-test**.** (D) Phase contrast time-lapse stills of control and pacsin 2-depleted cells undergoing cell division. Yellow arrowheads indicate plasma membrane blebs. Time is in minutes from the last frame of metaphase. Scale bar, 10 µm. (E) Quantification of cell division phenotypes in control versus pascsin 2-depleted cells. Bars represent the mean of 3 experiments with 100-120 cells analyzed per condition per experiment. Error bars represent SEM. ***$p<0.0001$, Chi-squared analysis.

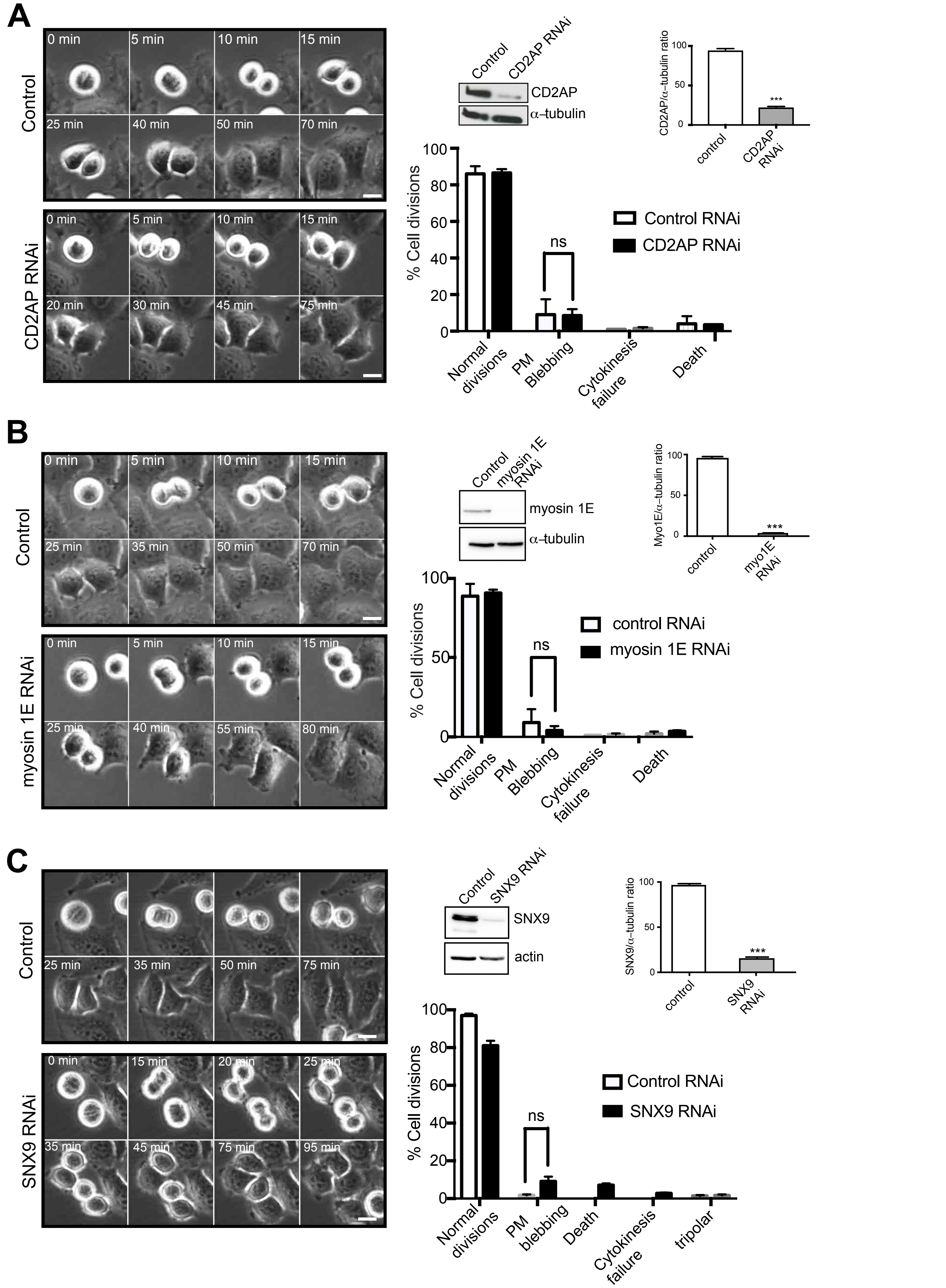

Figure S5. Knockdown of CD2AP, myosin1E and SNX9 do not cause cortical blebbing in cytokinesis. Related to Figure 7. Cells were depleted of CD2AP (A), myosin1E (B), or SNX9 (C) and cytokinesis analyzed. Left panels show phase contrast time-lapse stills of the dividing cells. Scale bars, 10 µm. Right panels show quantification of cell divisions displaying the indicated phenotypes (top) and Western blots indicating the efficiency RNAi-mediated depletion of the indicated proteins (left) and quantitation of the relative protein abundance following depletion. Quantitation of the relative protein abundance following depletion.Values are means ± SEM of 3 independent experiments each done in triplicate, ***$p<0.0001$, Student's t-test**.** α-tubulin and actin are used as loading controls.

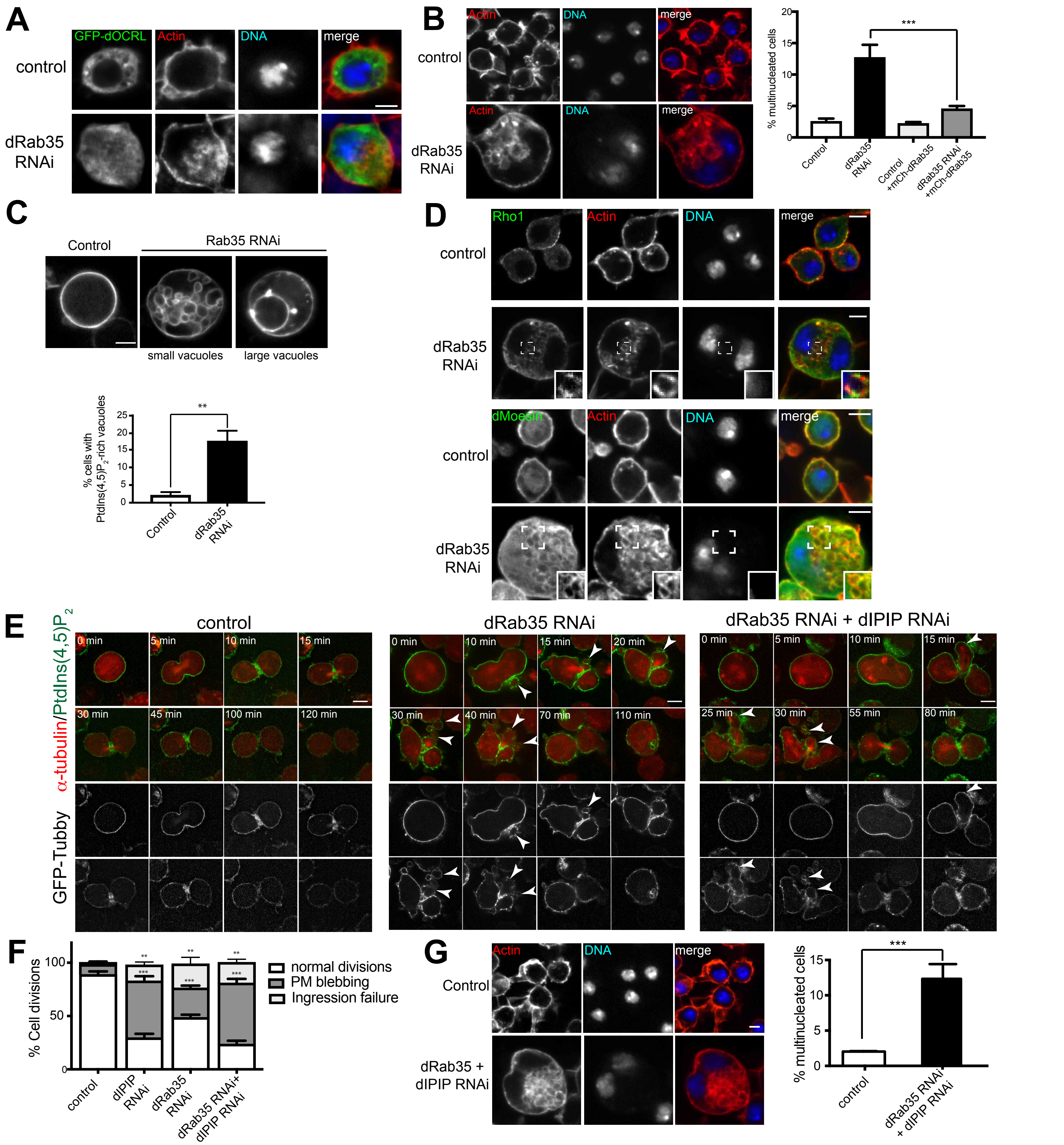

Figure S6. Knockdown of dRab35 phenocopies the cytokinesis phenotypes of dIPIP and dOCRL. Related to Fig7. (A) Localization of GFP-dOCRL G365E phosphatase-dead (green) in control or dRab35-depleted S2 cells stained with phalloidin for F-actin (red) and Hoescht 333258 (DNA, blue). (B) RNAi-treated S2 cells were stained with phalloidin (red) and Hoechst 33258 (blue). Quantification of multinucleation. Bars represent mean±SEM of 3 independent experiments with ~450 cells per experiment. ***p<0.0001 , Student's t-test. (C) Top, live stills of control or dRab35-depleted S2 cells stably expressing GFP-Tubby. Scale bar, 5 µm. Bottom, Percentage of cells with PtdIns(4,5)2-rich vacuoles. Bars represent mean ± SEM of 3 independent experiments with at least 200 cells analyzed per condition per experiment. **p<0.005, Student's t-test. (D). RNAi-treated S2 cells were labelled with anti-Rho1 or anti-dMoesin (green), phalloidin (red) and Hoechst 33258 (blue). (E) Selected time-lapse stills showing dynamics of GFP-Tubby during cell division. White arrowheads show membrane blebs. Time is in minutes from the last frame of metaphase. (F) Quantification of cell division phenotypes. Stacked bars represent the mean ± SEM from 3 experiments with 50-80 cells per experiment. **p<0.005, ***p<0.0005 , Chi-squared analysis. (G) Left, dRab35 and dIPIP RNAi-treated S2 cells were stained with phalloidin (red) and Hoechst 33258 (blue). Quantification of multinucleation. Bars represent mean± SEM of 3 independent experiments with ~450 cells per experiment. ***p<0.0001 , Student's t-test. Scale bars, 5 µm.
